# Sex differences and testosterone interfere with the structure of the gut microbiota through the bile acid signaling pathway

**DOI:** 10.3389/fmicb.2024.1421608

**Published:** 2024-10-18

**Authors:** Xueqing Duan, Yinli Nie, Xin Xie, Qi Zhang, Chen Zhu, Han Zhu, Rui Chen, Jun Xu, Jinqiang Zhang, Changfu Yang, Qi Yu, Kun Cai, Yong Wang, Weiyi Tian

**Affiliations:** ^1^School of Basic Medical Sciences, Guizhou University of Traditional Chinese Medicine, Gui Yang, China; ^2^CAS-Key Laboratory of Synthetic Biology, CAS Center for Excellence in Molecular Plant Sciences, Institute of Plant Physiology and Ecology, Chinese Academy of Sciences, Shanghai, China

**Keywords:** sex differences, gut microbiota, composition, testosterone, bile acid signaling pathway

## Abstract

**Background:**

The gut microbiome has a significant impact on human wellness, contributing to the emergence and progression of a range of health issues including inflammatory and autoimmune conditions, metabolic disorders, cardiovascular problems, and psychiatric disorders. Notably, clinical observations have revealed that these illnesses can display differences in incidence and presentation between genders. The present study aimed to evaluate whether the composition of gut microbiota is associated with sex-specific differences and to elucidate the mechanism.

**Methods:**

16S-rRNA-sequencing technology, hormone analysis, gut microbiota transplantation, gonadectomy, and hormone treatment were employed to investigate the correlation between the gut microbiome and sex or sex hormones. Meanwhile, genes and proteins involved bile acid signaling pathway were analyzed both in the liver and ileum tissues.

**Results:**

The composition and diversity of the microbiota from the jejunum and feces and the level of sex hormones in the serum differed between the sexes in young and middle-aged Sprague Dawley (SD) rats. However, no similar phenomenon was found in geriatric rats. Interestingly, whether in young, middle-aged, or old rats, the composition of the microbiota and bacterial diversity differed between the jejunum and feces in rats. Gut microbiota transplantation, gonadectomy, and hormone replacement also suggested that hormones, particularly testosterone (T), influenced the composition of the gut microbiota in rats. Meanwhile, the mRNA and protein level of genes involved bile acid signaling pathway (specifically *SHP*, *FXR*, *CYP7A1*, and *ASBT*) exhibited gender-specific differences, and T may play a significant role in mediating the expression of this pathway.

**Conclusion:**

Sex-specific differences in the structure of the gut microbiota are mediated by T through the bile acid signaling pathway, pointing to potential targets for disease prevention and management techniques by indicating that sex differences and T levels may alter the composition of the gut microbiota via the bile acid signaling pathway.

## Highlights

- In young and adult SD rats, the composition of the gut microbiota, T levels, and mRNA and protein levels of genes related to the bile acid signaling pathway were significantly gender-specific, with T levels in male rats significantly higher than that in females.
- Gonadectomy, which involves the removal of testicles in male rats and ovaries in female rats, along with hormone replacement therapy (involving testosterone for male rats with testicular removal and estradiol for female rats with ovary removal), has been found to impact the gut microbiota, as well as the mRNA and protein levels of genes associated with the bile acid signaling pathway through mediation by T.
- The “T-bile acid-gut microbiota” axis, which highlights the role of T in modulating the gut microbiota through bile acid signaling, suggests promising avenues for disease prevention and management strategies.

## Introduction

The animal gut serves as a sophisticated digestive system, housing a diverse array of microbial communities consisting of bacteria, archaea, fungi, protozoa, and viruses (Hallen-Adams and Suhr, 2017). Studies have demonstrated a significant and mutually advantageous symbiotic relationship between the intestinal flora and the host. The host gut microbiota plays a crucial role in various cellular functions including immunity, anti-tumor activities, anti-aging properties, detoxification, and nutrition (Thaiss et al., 2016; Yu et al., 2024; Wang et al., 2024; Shamjana et al., 2024; Li et al., 2024). This highlights the essential contribution of gut microbiota to overall host health and well-being.

The absorption and digestion of food and drugs in the human body occur in the gastrointestinal tract, leading to a significant focus on the relationship between these substances and intestinal flora. Contemporary research has revealed that the primary modes of interaction between food or drugs and intestinal flora encompass the mutual regulation of intestinal flora structure by food or drugs, the regulation of intestinal flora metabolism by food or drugs, and the transformation metabolism of food or drug components by the intestinal flora (Qu et al., 2019; Wu and Tan, 2019; Zhang et al., 2020; Vich Vila et al., 2020). The bioactive metabolites produced by gut microorganisms, such as bile acids (Chávez-Talavera et al., 2017; He et al., 2024), trimethylamine-N-oxide (TMAO; Wang et al., 2011; Wang et al., 2024a), branched-chain amino acids (BCAAs; Solon-Biet et al., 2019), vitamins (Brunkwall and Orho-Melander, 2017), and short-chain fatty acids (SCFAs; Zhao et al., 2018), play a crucial role in maintaining host health. These metabolites are essential for various physiological functions and overall well-being. For instance, The elevation of total bile acid levels is widely recognized as a crucial pathogenic and pathological feature of insulin resistance and type 2 diabetes mellitus (T2DM; Kaska et al., 2016; Zhu X. et al., 2024). Studies have demonstrated that the gut microbiota can perturb intercellular communication by modifying the molecular configuration of bile acids, thereby affecting the circulation of bile acids between the liver and intestines and subsequently influencing the onset and progression of T2DM (Gao et al., 2022; Tian et al., 2022). In their 2024 study, Lu et al. found a positive linear association between elevated levels of unconjugated bile acids, particularly secondary bile acids derived from gut microbiota, and increased risk of cardiovascular disease (CVD) in individuals. Zhao et al. (2018) also found that dietary fiber promotes the increase of intestinal flora that produces short-chain fatty acids, maintains blood glucose homeostasis by increasing Glucagon peptide 1(GLP-1) levels, and delays the progression of T2DM. In addition, small adjustments in BCAA metabolism have been implicated in playing a role in a wide range of prevalent diseases, such as diabetes, cancer, and heart failure (Gao and Hou, 2023; Kang et al., 2024; Wang et al., 2024b). Research has also shown that a deficiency in vitamin D is linked to a higher risk of developing T2DM, metabolic syndrome (MS), and CVD (Renke et al., 2023; Zhang et al., 2024; Hu and Yang, 2024). As such, gut dysbiosis has become an important target for the treatment of cardiovascular diseases, metabolic diseases, malignant tumors, and dementia (Donaldson et al., 2018; Rothhammer et al., 2018; Buffa et al., 2022; Wong and Yu, 2023; Luqman et al., 2024).

Numerous studies have shown that the gut microbiota is influenced by multiple factors, including host genetics, diet, medications, age, environment, and host gender (Goodrich et al., 2014; Kolodziejczyk et al., 2019; Vich Vila et al., 2020; Rudolph et al., 2022; Wu et al., 2024; Rio et al., 2024). Clinical studies have revealed that individuals of different genders exhibit variations in disease prevalence, age of onset, diagnosis, treatment methods, etc., which are linked to differences in sex hormones, genes, and behavior. The age-adjusted incidence rate (AIR) demonstrates higher rates in men for afflictions like tumors and respiratory diseases, while it is higher in women for conditions such as endocrine and metabolic disorders (Westergaard et al., 2019). Similarly, the composition of gut microbiota may be influenced by sex, and the variations in gut microbiota composition between genders may be associated with the levels of sex hormones (Org et al., 2016; Hansen et al., 2019; Shin et al., 2019; Santos-Marcos et al., 2023; Le Bras, 2024). For example, in pigs, the gender of the host significantly influences the phylogenetic composition of the gut bacterial community (Xiao et al., 2016). Takagi et al. (2019) observed substantial disparities in the composition of the intestinal flora between male and female subjects. Specifically, they noted elevated levels of Prevotella, Macromonas, Fusobacterium, and Micrococcus in the digestive tracts of males. At the same time, the relative abundance of Bifidobacterium, Rumenococcus, and Akkermansia was higher in females. Gender disparities in the gut microbiome play a crucial role in shaping host metabolism (Xie et al., 2017; Kadyan et al., 2023), influencing the regulation of autoimmunity (Markle et al., 2013), and determining responses to various diseases (Hokanson et al., 2024). The study conducted by Down et al. (1983) concluded that sex hormones impact bile acid metabolism. Robust research indicates that metabolites dependent on the gut microbiome have the potential to interact with significant biological pathways regulated by sex hormones. These pathways include toll-like receptors and flavin monooxygenase signaling (Razavi et al., 2019). Nevertheless, the precise mechanisms underlying the interplay between gender, gut microbiota, and disease pathology remain uncertain, and there is a notable absence of clinical cases focused on disease prevention and treatment strategies targeting gender-dependent gut microbiota.

In addition, several cross-population studies have compared the gut microbiome of females with males in humans or mice (Schnorr et al., 2014; Org et al., 2016; Hansen et al., 2019; Santos-Marcos et al., 2023; Ortiz-Alvarez de la Campa et al., 2024). However, there is no consensus on whether the microbiome is sex-related. Some research reports suggest that although sex differences influence physiology and behavior, sex has been shown to have no or limited impact on the intestinal flora (Lay et al., 2005; Kovacs et al., 2011; Human Microbiome Project Consortium, 2012). Previous studies have found differences in the intestinal flora between sexes only in certain areas or genotypes (Org et al., 2016; Hansen et al., 2019). Other studies have suggested that there are significant differences in the gut microbiota composition between sexes and that the composition of the gut flora can influence sex hormone levels (Qin et al., 2010; Markle et al., 2013; Bolnick et al., 2014; Shastri et al., 2015; Lledós et al., 2023; Rio et al., 2024). However, the mechanism underlying sex hormone influence on gut microbiota is not yet clear.

Our study employed 16S-rRNA-sequencing technology, hormone analysis, gut microbiota transplantation, gonadectomy, and hormone treatment to investigate the correlation between the gut microbiome and sex or sex hormones. We observed that the composition of the microbiota from the jejunum and feces, bacterial diversity per individual, and the levels of estradiol (E2) and testosterone (T) from serum differed between male and female adult SD rats. In younger rats, there were significant differences in the composition of the jejunal microbiota and serum levels of E2 and T between sexes, whereas, in geriatric rats, there were no significant differences in the composition of the jejunal and fecal microbiota and serum T levels between the sexes. Interestingly, whether in young, middle-aged, or old rats, the composition of the microbiota and bacterial diversity differed between the jejunum and feces. Gut microbiota transplantation, as well as gonadectomy (Testicle extraction in male rats and ovaries extraction in female rats) and hormone replacement therapy (Male rats with testicular removal were treated with T, and female rats with ovary removal were treated with E2), also suggested that hormones, particularly T, influenced the composition of the gut microbiota in rats. mRNA and protein analyses showed that genes, such as small heterodimer partner (*SHP*), farnesoid X receptor (*FXR*), cholesterol 7α-hydroxylase (*CYP7A1*), and apical Na^+^-bile acid transport (*ASBT*), involved in the bile acid signaling pathway exhibited gender-specific differences and that T may affect the expression of genes involved in the bile acid signaling pathway. Thus, altering metabolic signaling pathways is one potential way T influences gut microbiota composition.

## Materials and methods

### Sample collection

Specific-pathogen free (SPF) SD (Sprague Dawley; SD) female and male rats were used to determine age-and gender-specific differences in the gut microbiota. The rats were divided into three groups: 20 rats (*n* = 10 rats/sex) were in the young age group, which were purchased at the age of 1 week and fed until 2 weeks of age; 20 rats (*n* = 10/sex) were in the middle age group—purchased at 1 week of age and fed until 8 weeks of age; and 18 rats (*n* = 9 rats/sex) were in the old group, which were purchased at 1 week of age and fed until 92 weeks of age (old). For gonadectomy, sex hormone therapy, and fecal microbiota transplantation, SD rats were purchased at 7 weeks of age and fed until 8 weeks of age (middle age). All SPF animals were purchased from Shanghai Kilton Laboratory Animal Technology Co., Ltd. [license No. SCXK (shanghai) 2017–0005] and were maintained on a 12-h light/dark schedule in separate cages in a room with a temperature of 23 ± 2°C and humidity of 40–70%. The animals had access to chow and water *ad libitum*. Additionally, the rats received regular replacement of their bedding and sterile drinking water. Rats were euthanized by exsanguination under anesthesia by intraperitoneal injection of urethane (1,000 mg/kg; Hrapkiewicz et al., 1998).

Liver tissue, ileum, jejunum, and fecal samples were snap-frozen with liquid nitrogen and stored at −80°C. The blood samples for hormonal measurements were obtained from the heart following deep anesthesia. Blood samples were centrifuged at 3000 rpm for 10 min at 4°C, and the supernatant (serum) was collected and stored at −80°C until use. Unless otherwise specified, the animals were raised at the Experimental Animal Center of Guizhou University of Traditional Chinese Medicine [License No. SYXK (Guizhou) 2021–0005]. All animal-related operations involved in this study have been approved by the Animal Ethics Committee of Guizhou University of Traditional Chinese Medicine (Approval No. 2020002).

### 16S rRNA gene sequencing and analysis

Microbial DNA was extracted, and the 16S rRNA gene of isolated DNA was sequenced by the Illumina MiSeq platform as described in a previous study (Org et al., 2015). The open-source pipeline Quantitative Insights Into Microbial Ecology (QIIME) version 1.7.0 was used to perform de-multiplexing 16S rRNA gene sequences, quality control, and operational taxonomic unit (OTU) binning (Bokulich et al., 2013). Sequences were binned into OTUs based on 97% identity using UCLUST31 against the Greengenes reference database (McDonald et al., 2012). In QIIME, the summarized taxa function was used to define microbial composition at each taxonomic level. Beta diversity (Bray-Curtis) dissimilarity and Principal Coordinates Analysis (PCoA) were evaluated using the QIIME program. Comparison of group differences in the microbiota within and between intestine (jejunum and feces) and gender (male and female) was performed using the Adonis function for permutational multivariate analysis of variance (PERMANOVA) and multivariate analysis of variance (MANOVA). Significant differences in taxa modulated by sex and specific intestinal location were determined by linear discriminant analysis effect size (LefSe; Org et al., 2015). The above work related to 16S rRNA sequencing was completed by Shanghai Zhejiang Bioengineering Co., LTD.

### Sex hormonal analysis

A rat-specific ELISA kit was used to measure blood sera levels of E2 and T (Wuhan Aidi Anti-biological Technology Co., LTD., Batch no. CSB-E11162r).

### Gonadectomy and sex hormone therapy

A total of 32, 1-week-old SD rats (16 females and 16 males) were randomly divided into a female sham group (F-sham), male sham group (M-sham), female operation group (OVX), male operation group (CAS), female operation + hormone group (OVX + E2), and male operation + hormone group (CAS + T), with five or six rats in each group. The operations related to gonadectomy and sex hormone therapy were performed based on previous protocols (Benedek et al., 2017; van der Giessen et al., 2019). Male and female SD strain rats were subjected to gonadectomy at 1 week of age under isoflurane anesthesia. In male rats, bilateral incisions were made in the scrotal region, the testes were removed, and the incisions were closed with wound clips. In female rats, the ovaries were removed through an incision just below the rib cage, followed by suturing of the muscle layer and closure of the incision with wound clips. Sham-operated control rats underwent the same incisions and closures, with brief manipulation of the gonads but without removal. The OVX + E2 group received estradiol valerate tablets via intragastric administration, with a calculated daily dosage of 1 mg for adults with a body weight of 60 kg. The conversion coefficient of administration between rats and adults was 6.3 times, and the dose of estradiol valerate tablets was 0.1 mg/kg, with sterile water configuration and an intragastric volume of 1 mL/100 g. The CAS + T group was administered testosterone undecanoate soft gel via intragastric administration, with a dosage calculated as 160 mg daily for a 60 kg adult body weight. The conversion coefficient between rats and adults was 6.3 times, and the dosage of testosterone undecanoate soft gel was 16.8 mg/kg, configured with sterile water. The intragastric volume was 1 mL/100 g. The M-sham and F-sham groups, as well as the OVX and CAS groups, were given the same volume of sterile water once a day. After 7 weeks, at 8 weeks of age, the rats were examined for changes in their gut microbiota in each group. The conditions under which the gut microbiota were sampled were consistent with the previous experiment.

### Fecal microbiota transplantation

A total of 20, 1-week-old female and male SD rats were fed for 2 weeks and randomly divided into female (F) and male (M) blank control group (donor), and female (M → F) and male (F → M) transplantation group (recipient), with four or five rats in each group. The recipient group was administered an antibiotic mixture for pseudo-sterile rat modeling, and the donor group was administered an equal volume of sterile water (Kolodziejczyk et al., 2019). The operations related to fecal microbiota transplantation were performed as previously reported by Zhao et al. (2018). Fecal samples were obtained from rats that had been fasted for 12 h. Each group provided 80–100 mg of feces, which was then mixed with 600 uL of PBS buffer. The fecal material was thoroughly suspended by vortexing for 5 min and allowed to settle by gravity for an additional 5 min. Following centrifugation at 2500 rpm for 1 min, the clear supernatant was carefully transferred to a clean tube, and an equal volume of 20% (w/v) skimmed milk (LP0031, Oxoid, United Kingdom) was added. The inoculum was freshly prepared on the day of the experiment. Regular bacteriological examinations of feces, food, and fillings were conducted. Transplantation was carried out at 8:00 a.m. once daily. After 6 weeks, at 8 weeks of age, the rats were examined for changes in their gut microbiota in each group. The conditions under which the gut microbiota were sampled were consistent with the previous experiment.

### Quantitative real-time PCR

qRT-PCR reactions were carried out in an optical 96-well plate in a CFX96 instrument (Bio-Rad, United States). The primers used for quantitative PCR analysis are listed in Supplementary Table S10. mRNA was extracted from the liver and ileum of SPF SD female and male rats at the age of 12 days (young), 8 weeks (middle age), and 92 weeks (old) following common feeding. Additionally, mRNA was extracted from the same organs at the age of 8 weeks (middle age) after fecal microbiota transplantation and hormone therapy. The RNA extraction was performed using a bacterial RNA kit (Omega, Lot no. R6828-02), and complementary DNA (cDNA) was synthesized from 1 μg of the total RNA utilizing a reverse transcription kit (Beijing Kang Wei Century Biotechnology Co., LTD., Batch no. CW2582M) in a total volume of 20 μL. Subsequently, the transcription levels of genes associated with the bile acid signaling pathway were determined by qRT-PCR. The qRT-PCR reaction comprised 25 μL 2× TransStartTM SYBR Top Green qPCR SuperMix (Beijing Kang Wei Century Biotechnology Co., LTD., Batch no. CW0957M), 1 μL forward primer (10 mM), 1 μL reverse primer (10 mM), and 1 μL of 1:10 diluted cDNAs, with ultrapure water used to make up the total volume to 50 μL. The qRT-PCR was conducted under the following conditions: 1 cycle at 95°C for 10 min, followed by 40 cycles at 95°C for 15 s and 60°C for 1 min. The data analysis was performed using Bio-Rad CFX Manager v.2.1 software, with the expression levels of different genes normalized by *β*-actin and GAPDH. The methods and data evaluation of qRT-PCR analysis were executed following the approach reported by Pfaffl (2001). The mRNA levels of individual genes were normalized and calculated using the 2^ΔΔCT^ method, where the relative expression level of a target gene is represented in a sample versus a control in comparison to a reference gene. ΔCt target is the Ct value of the control - sample of the target gene transcript. ΔCt reference is the Ct value of the control - sample of reference gene transcript. The relative expression level of a target gene = 2ΔCt target/2ΔCt reference = 2ΔΔCT. It is noteworthy that three technical replicates were carried out for each cDNA sample, with more than three biological replicates (derived from different rats growing under the same conditions) performed for sample repeat analyses.

### Western blots

Liver tissue (80–90 mg) or ileum tissue (20–25 mg) was lysed in 1000 μL or 300 μL RIPA lysis buffer (Epizyme PC101), respectively, containing 1% territory NP-40 solution, 1% sodium deoxycholate, 0.1% sodium dodecyl sulfate, and 1% protease inhibitor diluted in Tris base buffered with HCl (pH 7.4) before being sonicated. The homogenate was centrifuged at 12,000 rpm for 5 min at −4°C as previously described (Alves et al., 2011), and the supernatants were transferred to new tubes and stored at −80°C until use. Protein concentrations were determined according to the BCA Protein Quantitation Kit (ZJ 101). Western blotting was performed as a routine, with proteins separated on 12% SDS-polyacrylamide gels (Bio-Rad) at 80 V for 90 min and then transferred to polyvinylidene fluoride membranes (Bio-Rad) at 120 V for 90 min. The membranes were blocked with a solution of 5% non-fat dry milk and 0.05% Tween-20 (TBS-T; Sigma) for 15 min. Subsequently, the primary antibodies targeting eight putative constitutively expressed proteins were added and left to incubate at 4°C overnight. After incubation, the membrane was washed with 1 × TBST using an oscillating method for 10 min, repeated 3 times. Following this, a secondary antibody (HRP-conjugated Goat Polyclonal antibody) was applied and incubated at 37°C for 1 h, and the membrane was washed again with 1 × TBST using an oscillating method for 10 min, repeated 3 times. The working concentrations and relevant information of all antibodies used in this study can be found in Supplementary Table S11. The ECL luminescent developer was prepared and the PVDF film was completely immersed in the ECL luminescent developer in a dark environment, with development lasting 30 s. Immunoblots were developed with Luminata (Millipore) as per the manufacturer’s instructions, and densitometric results were analyzed using Image J software. Coefficients of variance were calculated by determining the ratio between standard deviation and mean. Band densities of the target protein were normalized using *β*-actin Rabbit mAb. The antibodies utilized in this study were procured from Shanghai Uning Life Science and Technology Co., LTD.

### Immunofluorescence

Liver sections were incubated with CYP7A1, FXR, and SHP (rabbit, Santa Cruz, 1: 50) at 4°C for 12 h, and then incubated with an Alexa fluor 488-conjugated goat anti-rabbit IgG and DAPI for 90 min and 10 min, respectively.

### Statistical analysis

GraphPad Prism V8.0 (San Diego, California, United States) was used for analysis and graph preparation. For the graph data related to 16S rRNA gene sequencing, the results are expressed as mean ± SD and statistical analyses were performed using the two-tailed non-parametric Mann–Whitney U test or Kruskal-Wallis test with Benjamini-Hochberg FDR multiple comparison. Statistical significance of sample grouping for beta-diversity analysis was performed using the Permanova method (999 permutations) and multivariate analysis of variance (MANOVA). The remaining data was shown as means ± SEM. Differences were assessed by the Students’ t-test or a one-way ANOVA, and denoted as follows: *p < 0.05; **p < 0.01; ***p < 0.001; and ns *p* > 0.05.

## Results

### Gender-specific gut microbiota composition in middle-aged rats

**Figure 1:**
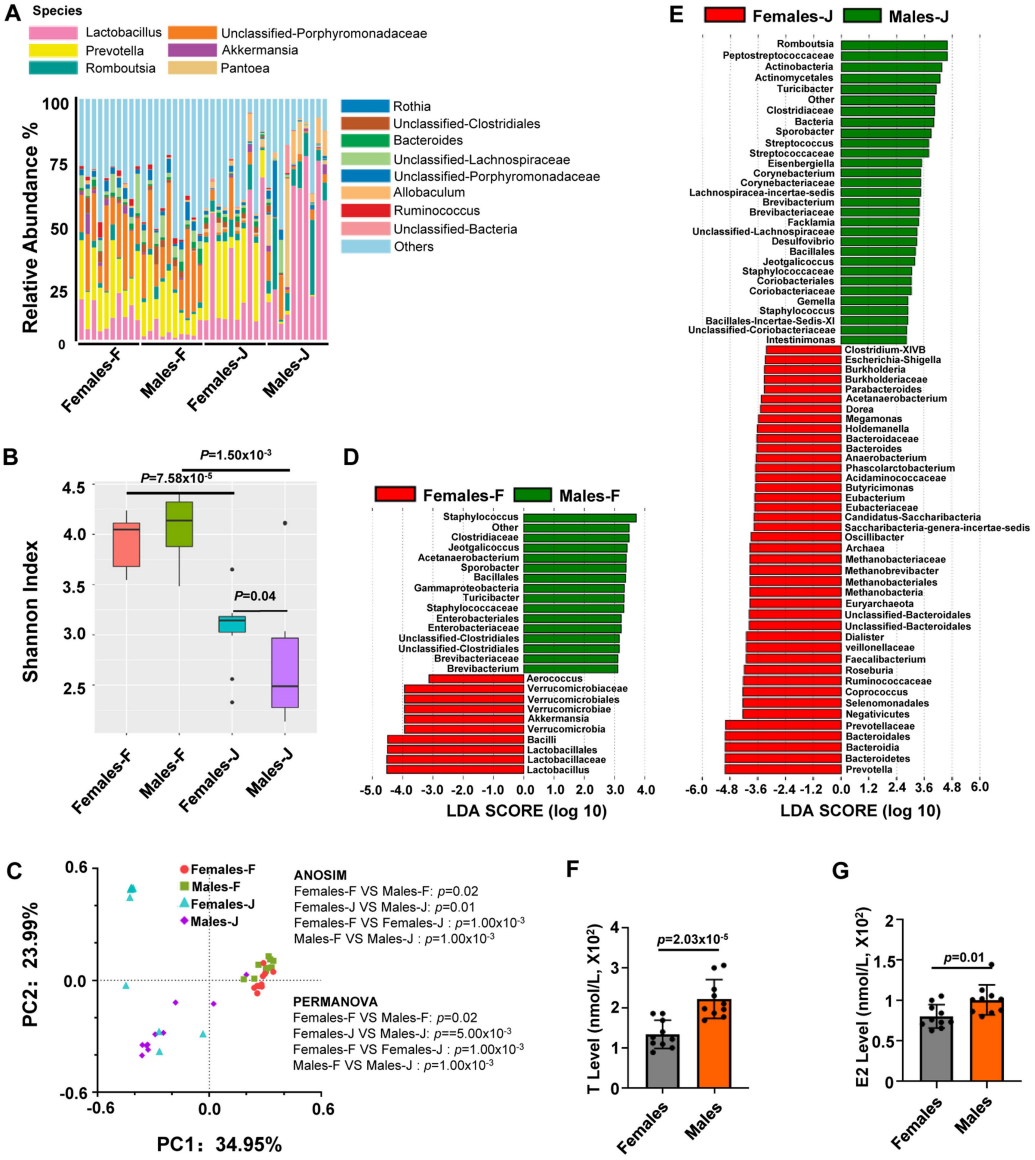
Sex differences in the gut microbiota composition and hormone level of middle-aged rats. (A) The relative abundance per individual for fecal and jejunal samples from middle-aged female and male rats, shown for the bacterial taxonomic rank of species; (B) within group mean α diversity, *p* values from Mann–Whitney U test; (C) Principal coordinate analysis (PCoA) plot of Bray-Curtis distances, analysis of similarity (ANOSIM) and permutational multivariate analysis of variance (PERMANOVA) of the microbial communities between sexes. *p* values from ANOSIM and PERMANOVA are shown for sex. (D,E) Linear discriminant analysis (LDA) of gut microbiota composition of rats. The LDA coupled with effect size measurements identified the most differentially abundant taxa between females and males in fecal or jejunal samples from rats, all taxa detected at the phylum to genus level. (F,G) The analysis for the levels of estradiol (E2) and testosterone (T) from serum samples. Student’s *t* tests were used to generate the *p*-values. *n* = 10 per group; F stands for fecal samples; J stands for jejunal samples.

To assess whether the microbiome is sex-related, we compared the bacterial composition of fecal and jejunal samples from middle-aged female and male rats. We found significant sex differences in the composition of the microbiome from the jejunum and feces (Figure 1A). Fecal or jejunal samples varied in gut microbial *α*-diversity (bacterial diversity, or bacterial richness and evenness, within each group) between the sexes in rats, as quantified with the Shannon diversity index [a minimum of seven individuals per sex for the Mann–Whitney U (MWU) test; Figure 1B]. Principal coordinate analysis (PCoA) plot of Bray-Curtis distances of fecal and jejunal samples also showed clear patterns differentiating samples between males and females (Figure 1C). Moreover, analysis of similarity (ANOSIM) and permutational multivariate analysis of covariance (PERMANOVA) also indicated that a matrix of major PCoA axes was dependent on sex (Figure 1C; Supplementary Tables S1, S2). The above results suggested that the gut microbiota composition had significant gender specificity in SD rats.

To gain insights into this variation, we employed linear discriminant analysis (LDA) to pinpoint the specific genus-level taxa that exhibited sex-related changes in middle-aged rats. (Figures 1D,E). In the analysis of fecal samples, it was observed that several genera exhibited higher abundance in male rats compared to female rats. These genera included *Staphylococcus*, *Jeotgalicoccus*, *Acetanaerobacterium*, *Sporobacter*, *Turicibacter*, and *Brevibacterium*. Conversely, the abundance of *Aerococcus*, *Akkermansia*, and *Lactobacillus* genera in female rats was significantly higher than in males, as indicated in Figure 1D and Supplementary Table S3. In the jejunal samples, the abundance of certain genera such as *Romboutsia*, *Turicibacter*, *Sporobacter*, *Streptococcus*, *Eisenbergiella*, *Corynebacterium*, *Lachnospiracea-incertae-sedis*, *Brevibacterium*, *Facklamia*, *Desulfovibrio*, *Jeotgalicoccus*, *Gemella*, *Staphylococcus*, and *Intestinimonas* was significantly higher in male rats compared to female rats. On the other hand, the abundance of genera including *Clostridium-XIVB*, *Escherichia-Shigella*, *Burkholderia*, *Parabacteroides*, *Acetanaerobacterium*, *Dorea*, *Megamonas*, *Holdemanella*, *Bacteroides*, *Anaerobacterium*, *Phascolarctobacterium*, *Butyricimonas*, *Eubacterium*, *Candidatus-Saccharibacteria*, *Saccharibacteria-genera-incertae-sedis* was significantly higher in female rats than in male rats (Figure 1E; Supplementary Table S3).

### The effect of sex hormones on the microbiota

In a follow-up study, we examined the serum levels of T and E2 in males and females in middle-aged rats. We found that there were significant differences in both T and E2 levels between male and female rats (Figures 1F,G).

**Figure 2:**
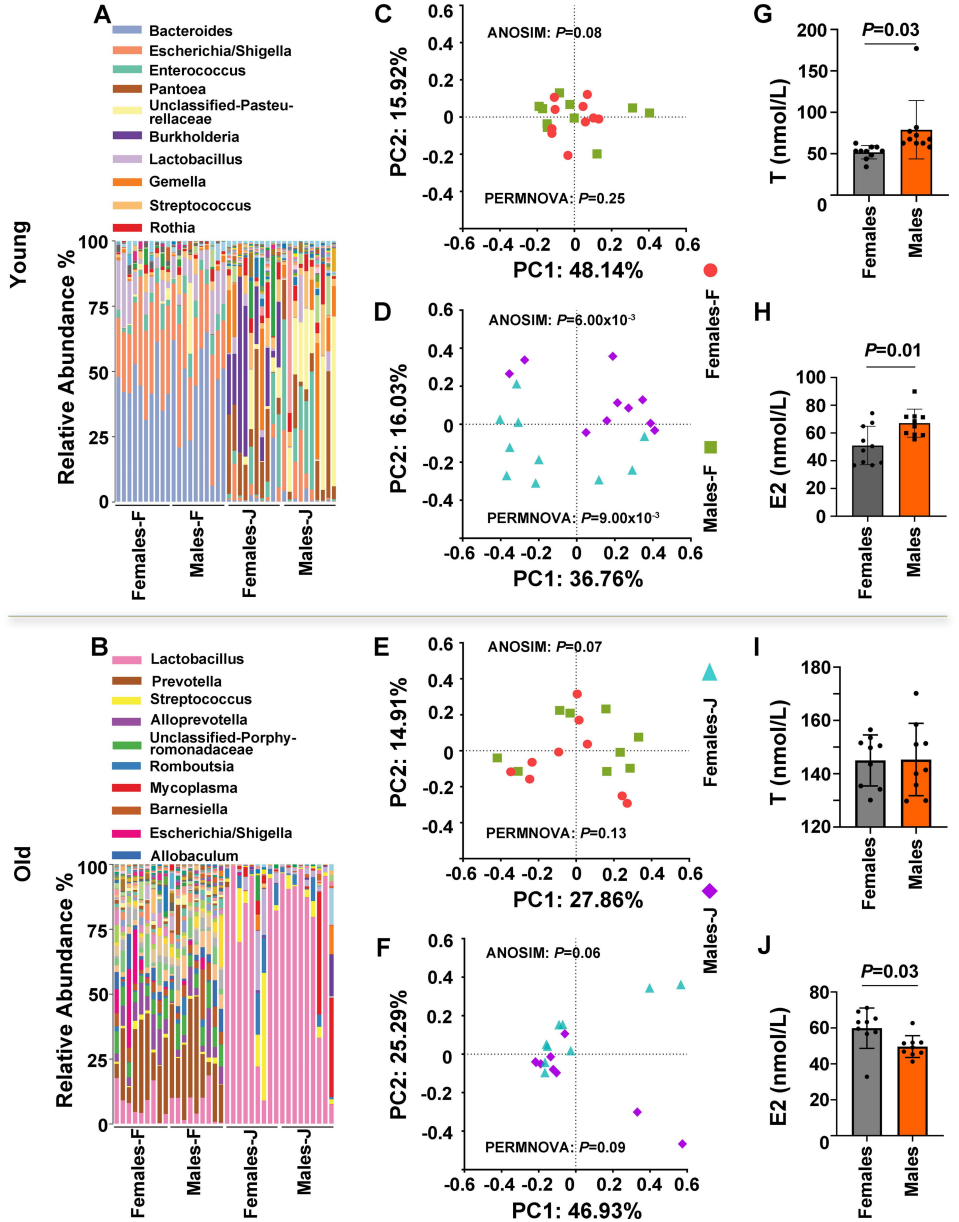
The gut microbiota composition and hormone level of young and old rats. (A,B) The relative abundance per individual for fecal and jejunal samples from female and male rats, shown for the bacterial taxonomic rank of genus. (C–F) PCoA plot of Bray-Curtis distances, ANOSIM, and PERMANOVA of the microbial communities between sexes. *p* values from ANOSIM and PERMANOVA are shown for sex. The relative analysis for fecal samples (C,E) and jejunal samples (D and F) from male and female rats. (G–J) Detection of serum testosterone (T) and estradiol (E2) levels in male and female rats. Student’s *t* tests were used to generate the *p*-values. Young and old rats were used in this experiment. F stands for fecal samples; J stands for jejunal samples; *n* = 9–10 per group.

To explore the causes of sex differences in the gut microbiota composition, we surveyed the bacterial composition of fecal and jejunal samples and tested the serum levels of T and E2 from young and old male and female rats (Figure 2). Our results indicated that although there were no significant differences in the microbiota composition of fecal samples from young rats between the sexes, there were significant gender differences in jejunal samples (Figure 2A). PCoA plots of Bray-Curtis distances of fecal and jejunal samples also indicated that no clear patterns differentiated fecal samples from males and females, whereas clear patterns differentiated jejunal samples from males and females in young rats (Figures 2C,D). ANOSIM and PERMANOVA were also used to test whether a matrix of major PCoA axes was dependent on sex. We observed that sex has significant effects on the microbiota of jejunal samples (ANOSIM: *p* = 6.00 × 10^−3^; PERMANOVA: *p* = 9.00 × 10^−3^), but no significant differences in fecal samples (ANOSIM: *p* = 0.08; PERMANOVA: *p* = 0.25) between sexes in young rats (Figures 2C,D; Supplementary Tables S4, S5). Additionally, we observed that both T and E2 levels were significantly different between the sexes in young rats (Figures 2G,H).

However, in the case of old rats, no significant differences in the microbiota composition of both fecal samples and jejunal samples were noted between the sexes (Figure 2B). A PCoA plot of Bray-Curtis distances of fecal and jejunal samples also indicated that no clear patterns differentiated fecal samples and jejunal samples between the sexes (Figures 2E,F). In addition, Both ANOSIM and PERMANOVA of the matrix of major PCoA axes showed no significant *β*-diversity distance between the sexes (Figures 2C,D; Supplementary Tables S4, S5). Interestingly, there were significant differences in E2 levels between the sexes in older rats (*p* = 0.03; Figure 2J), while there were no significant differences in T levels differences between the sexes (*p* > 0.05; Figure 2I). Based on the above observations, we inferred that sex hormones, particularly T, may affect the gut microbiota.

### Spatial specificity of the gut microbiota composition

In this study, we also compared the gut microbiota composition of fecal samples and jejunal samples from middle-aged female and male rats. Analysis of the taxonomic composition and Shannon diversity index of the gut microbiota indicated that there were significant differences in the microbiota composition between the jejunum and feces in both male and female rats (Figure 1A). Both PCoA of Bray-Curtis distances of fecal and jejunal samples from male and female rats, as well as ANOSIM and PERMANOVA of the matrix of major PCoA axes, indicated that there were significant β-diversity distances between fecal samples and jejunal samples in both male and female rats (Figures 1B,C; Supplementary Tables S1, S2). For middle-aged rats, we also performed Linear Discriminant Analysis (LDA) coupled with effect size measure (LefSe), which showed that there were significant differences in the microbiota composition between jejunum and feces in both male and female rats (Supplementary Figure S1).

To further identify the microbial taxa that accounted for the greatest differences between fecal samples and jejunal samples, we surveyed the bacterial composition of samples from young and old female and male rats (Supplementary Figure S2). Both *α*-diversity (as quantified with the Shannon diversity index; Supplementary Figures S2A,B) and β-diversity (as quantified with PCoA plot of Bray-Curtis distances; Supplementary Figures S2C,D) were significantly different between fecal samples and jejunal samples in both male and female rats, with the degree of compositional difference between samples assessed with ANOSIM and PERMANOVA test of Bray-Curtis distances (*p* = 1.00 × 10^−3^; Supplementary Figure S2C,D; Supplementary Tables S4, S5). Furthermore, we performed a cladogram coupled with effect size measure to identify specific taxa that changed between fecal samples and jejunal samples (LefSe, at the phylum to genus level; Supplementary Figure S3). In young rats, the results indicated that increases in jejunal samples from both females and males were in the phylum *Proteobacteria*, class *Epsilonproteobacteria*, and class *Alphaproteobacteria*—all of which appeared to drive overall differences between fecal samples and jejunal samples (Supplementary Figures S3A,B). In old rats, however, increases in jejunal samples from females in the class *Bacilli* and genus *Abiotropphia* appeared to drive overall differences between fecal and jejunal samples, and increases in jejunal samples from males in the class *Bacilli* and genus *Granulicatella* appeared to drive overall differences (Supplementary Figures S3C,D). In addition, we found that the abundance of the phylum *Bacteroidates* and genus *Bacteroides* in fecal samples was higher compared to jejunal samples in both females and males. This phenomenon was consistent with what was observed in middle-aged rats (Supplementary Figure S1). These results suggested that the composition of intestinal flora also has spatial specificity.

### T-mediated shifts in the microbiota

**Figure 3:**
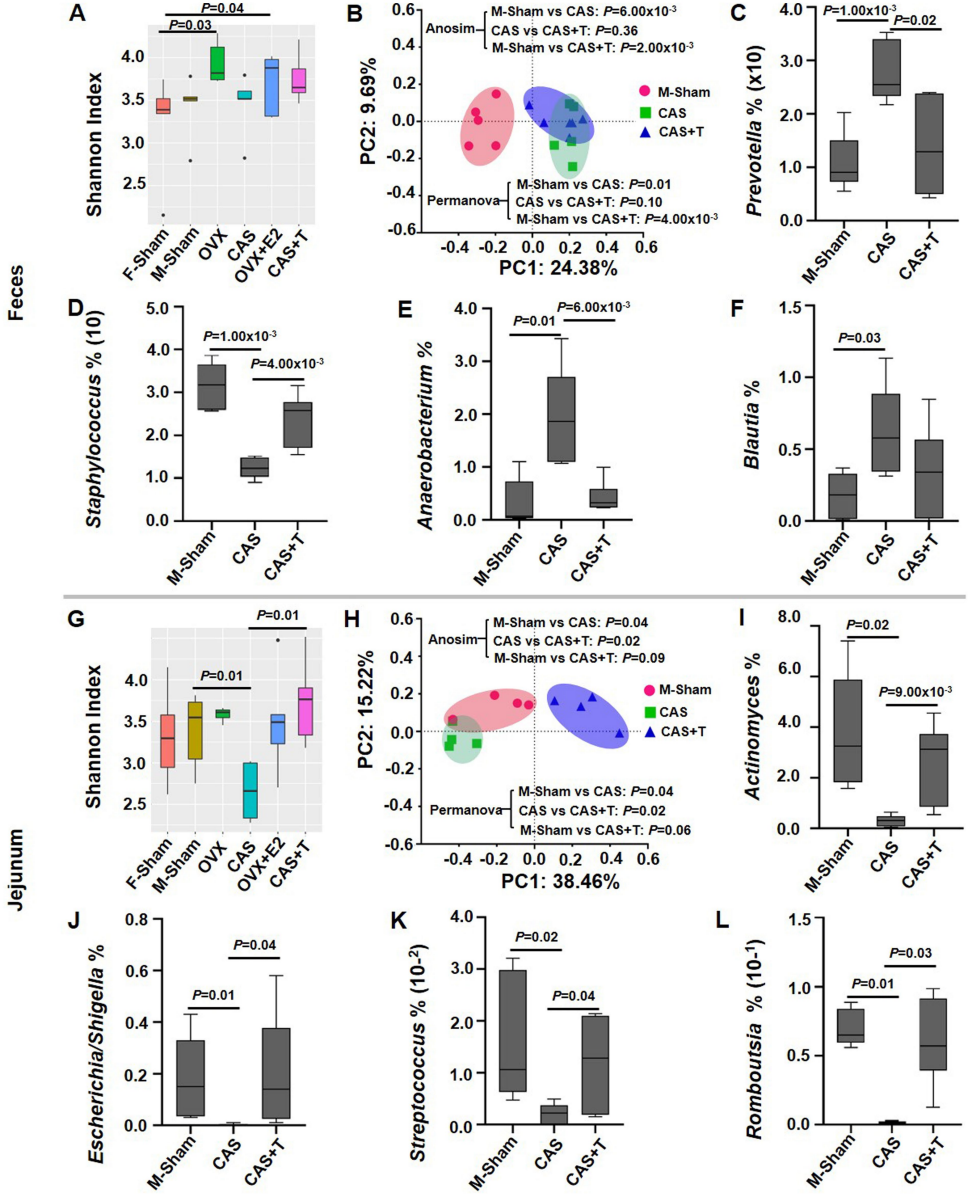
The effect of gonadectomy on the gut microbiota in middle-aged rats. (A) and (G) α diversity analyses of the microbial communities. (B) and (H) Principal coordinate analysis (PCoA) plot of Bray-Curtis distances of the microbial communities. (C–F) and (I–L) Box plots of relative abundance distributions per group for the taxa in male rats, where individuals are grouped by Sham, CAS, and CAS + T. In A–F, the data are derived from fecal samples; In G–L, the data are derived from jejunum samples. In the α--diversity analysis and analysis of relative abundance distributions per group for the taxa, the MWU test was used to generate the *p*-values. In PCoA analysis, *p* values from ANOSIM and PERMANOVA are shown. In this experiment, individuals were grouped by F-Sham, OVX, OVX + E2, M-Sham, CAS and CAS + T; *n* = 5–6 per group.

To confirm that sex differences were mediated by sex hormones in gut microbiota composition, we performed gonadectomy (GDX) with female and male rats and examined the gut microbiota composition after 8 weeks. The analysis of α-diversity and PCoA revealed that the gut microbiota composition had a clear separation between sham control and GDX in both females and males. Interestingly, in male rats, the analysis of α-diversity showed that administration of T following gonadectomy prevented significant gonadectomy-associated changes in the gut microbiota composition, particularly in the jejunum. Nevertheless, the PCoA analysis indicated that while there was still a distinct separation between the sham control and CAS + T group after T administration following gonadectomy, the introduction of T post-gonadectomy seemed to mitigate the pronounced alterations typically associated with gonadectomy on the composition of the gut microbiota. (Figures 3A,B,G,H; Supplementary Tables S6, S7). In the case of specific taxa (at the genus level), sham control males exhibited more abundant *Staphylococcus* and reduced abundance of *Prevotella*, *Anaerobacterium*, and *Blautia* than GDX males in feces, and the abundance of *Actinomyces*, *Escherichia*/*Shigella*, *Streptococcus*, and *Romboutsia* were more abundant in sham controls as compared to GDX males in the jejunum. Again, when administration of T following gonadectomy was performed, there was no significant difference in the abundance of these specific taxa between GDX + T males compared to sham controls (Figures 3C–F,I). However, this phenomenon did not occur in female rats (Figures 3A,G; Supplementary Figure S4; relevant results of microbial taxa are not shown).

Furthermore, we performed fecal microbiota transplantation (FMT) treatment with female and male rats and examined the gut microbiota composition after 12 weeks. Significant sex differences were observed in the composition of the microbiome (Supplementary Figure S5A; Supplementary Tables S8, S9). When fecal microbes from female rats were transplanted into males (F → M), the Bray-Curtis distances were very close between the F → M group and the male group. When fecal microbes from male rats were transplanted into females (M → F), the Bray-Curtis distances were significantly larger between the M → F group and the male or female groups (Supplementary Figure S5B). When analyzing the microbial taxa (at the genus level), the abundance of *Paraprevotella* and *Ruminococcus* in the F → M group was more prevalent in response to males, although the abundance of *Paraprevotella* and *Ruminococcus* was different between the sexes (Supplementary Figures S5C,D). In summary, the above results suggested that the gut microbiota correlates with both sex and sex hormones, particularly T, in rats.

### Gender-specific expression of genes related to bile acid signaling

**Figure 4:**
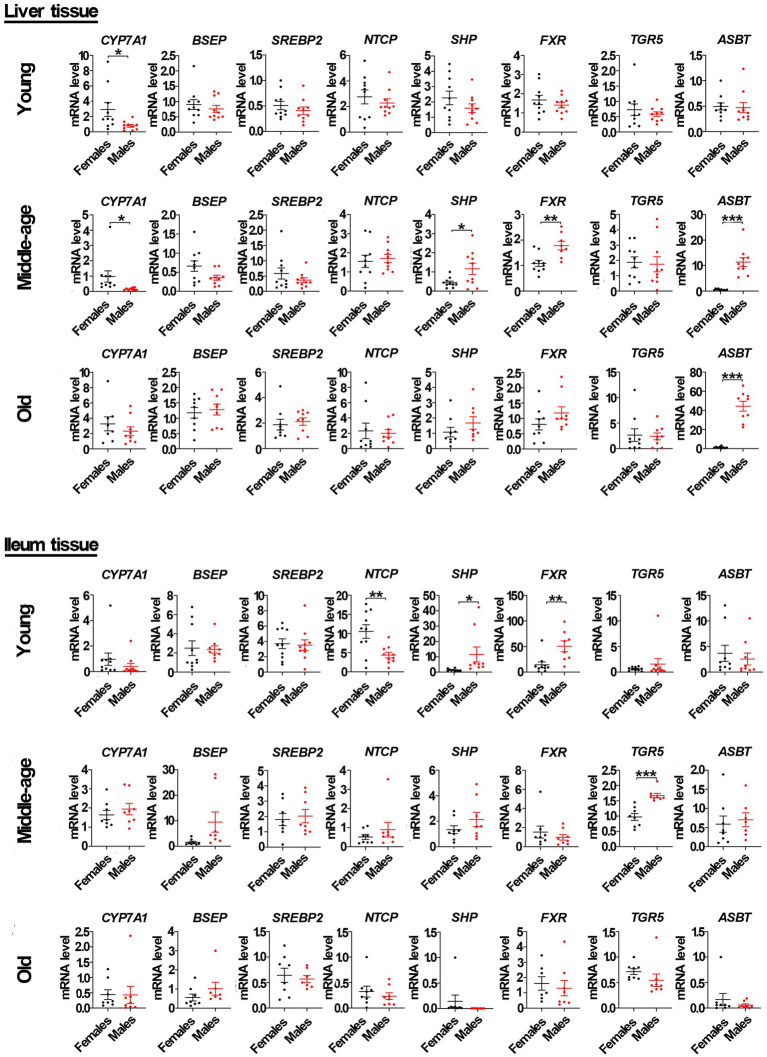
Gender-specific mRNA level of genes related to bile acid signaling pathway in liver and ileum tissue from young, middle-aged and old rats (females and males). Cholesterol 7α-hydroxylase (*CYP7A1*), bile salt export pump (*BSEP*), sterol regulatory element-binding proteins 2 (*SREBP2*), Na^+^/taurocholate co-transporting polypeptide (*NTCP*), small heterodimer partner (*SHP*), farnesoid X receptor (*FXR*), apical Na^+^-bile acid transport (*ASBT*), and G protein-coupled receptor (*TGR5*) relative mRNA abundances were determined by real-time PCR analysis, and relative gene pressions were normalized with *β-actin* and *GAPDH* (n = 9–10). Values are presented as the mean ± SEM of three technical repetitions. Differences were assessed by Student’s *t* tests and denoted as follows: *p < 0.05; **p < 0.01; ***p < 0.001; ns *p* > 0.05. Three biological replications were performed and all results were similar.

Recent studies have demonstrated that bile acids can affect the gut microbiota (Benedek et al., 2017; Zhao et al., 2018; Kolodziejczyk et al., 2019). Our study compared the mRNA level of genes related to bile acid signaling between the sexes among young, middle-aged, and old rats. In liver tissue from middle-aged rats, we determined that the mRNA levels of the *FXR*, *SHP*, and *ASBT* in male rats were significantly higher than that in females, while the mRNA level of *CYP7A1* in male rats was significantly lower than that in females. However, the mRNA levels of other genes, including bile salt export pump (*BSEP*), sterol regulatory element-binding proteins 2 (*SREBP2*), Na^+^/taurocholate co-transporting polypeptide (*NTCP*), and G protein-coupled receptor (*TGR5*), were not significantly different between the sexes. In the ileum, we found that the mRNA level of *TGR5* (but not *FXR*, *BSEP*, *SHP*, *SREBP2*, *NTCP*, *ASBT*, or *CYP7A1*) in middle-aged male rats was also significantly higher than that in middle-aged females. In the liver tissue from young male rats, only the mRNA level of *CYP7A1* was significantly lower as compared to female rats, but the mRNA levels of *FXR* and *SHP* in male rats were also significantly higher than that in females in the ileum. Moreover, the mRNA level of *NTCP* in male rats was significantly lower than that in the ileum of young female rats. However, no similar phenomenon occurred in both the liver and ileum tissues of old rats, except the mRNA level of *ASBT* being higher in male rats compared to females in liver tissues (Figure 4).

**Figure 5:**
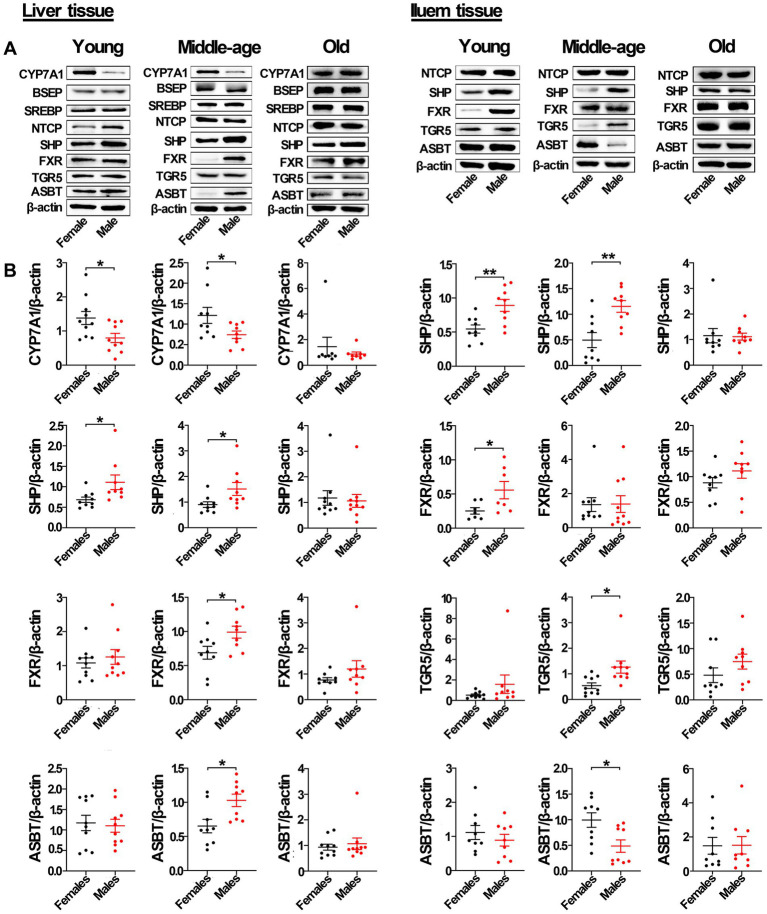
Gender-specific expression of proteins related to bile acid signaling pathway in liver and ileum tissue from young, middle-aged, and old rats (females and males). (A) CYP7A1, BSEP, SREBP2, NTCP, SHP, FXR, TGR5, and ASBT protein abundances were detected by western blot. (B) Quantification of CYP7A1, BSEP, SREBP2, NTCP, SHP, FXR, TGR5, and ASBT. The expression of target proteins were normalized to β-actin (n = 9–10). Values are presented as the mean ± SEM of three technical repetitions. Differences were assessed by Student’s *t* tests and denoted as follows: *p < 0.05; **p < 0.01; ***p < 0.001; ns *p* > 0.05. Three biological replications were performed and all results were similar.

As shown by Western blots analysis (Figure 5; Supplementary Figure S6), in liver tissue from middle-aged rats, the protein levels of FXR, SHP, and ASBT in male rats were also significantly higher than that in females, with the protein level of CYP7A1 in male rats significantly lower than that in females. However, the levels of other proteins, including BSEP, SREBP2, NTCP, and TGR5, were not significantly different between sexes. In the ileum from middle-aged rats, the protein levels of SHP and TGR5 in male rats were also significantly higher than that in females, while the protein level of ASBT in male rats was significantly lower than that in females. However, the protein levels of NTCP and FXR were not significantly different between the sexes. In liver tissue from young male rats, only the protein level of CYP7A1 was significantly lower than in female rats, with the protein levels of SHP and NTCP in male rats significantly higher than that in females. The levels of other proteins, including ASBT, FXR, BSEP, SREBP2, and TGR5, were not significantly different between the sexes. In ileum tissue from young rats, the protein levels of SHP and FXR (but not ASBT, NTCP, and TGR5) in male rats were also significantly higher than that in females. However, in old rats, the levels of proteins related to the bile acid signaling pathway, including CYP7A1, SHP, FXR, ASBT, BSEP, SREBP2, NTCP, and TGR5, were not significantly different between the sexes in liver tissue. Additionally, the levels of proteins in the bile acid signaling pathway, including SHP, FXR, ASBT, NTCP, and TGR5, were also not significantly different between the sexes in ileum tissue.

**Figure 6:**
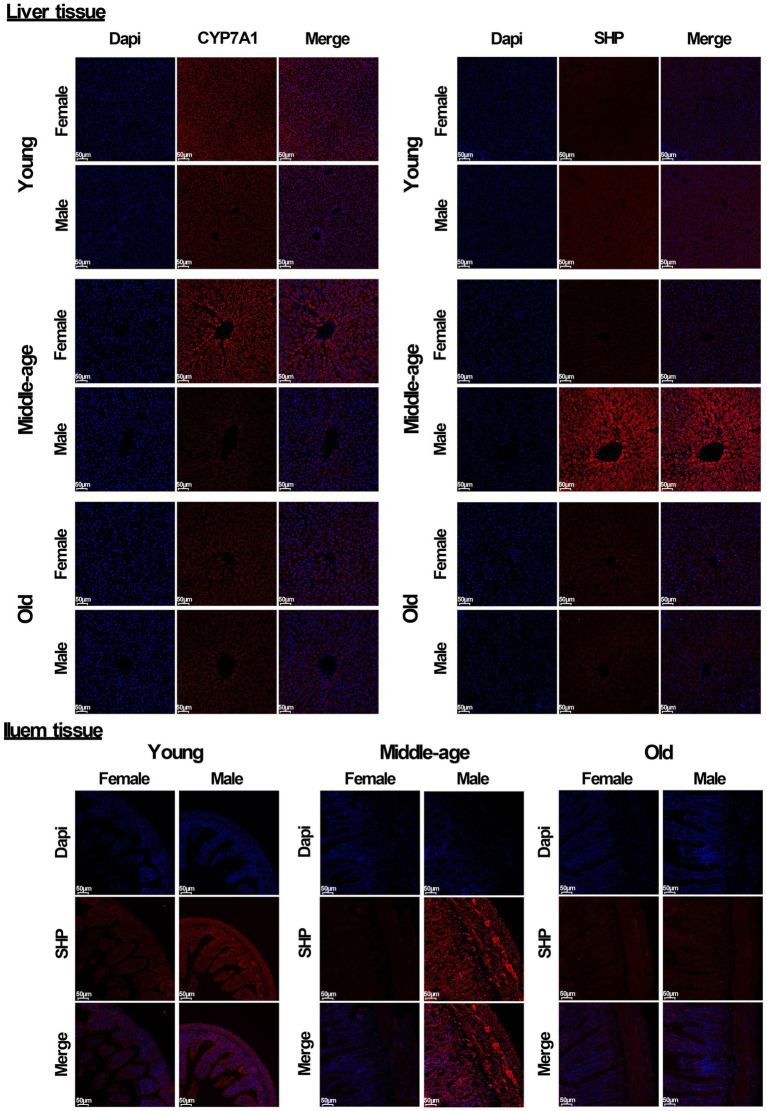
Immunofluorescent analysis of CYP7A1 and SHP (red) were performed in liver and ileum tissue from young, middle-aged and old rats (females and males). Nuclei were stained with DAPI (blue). Scale bar = 50 μm. three technical repetitions and three biological replications were performed and all results were similar.

In addition, immunofluorescent analysis of liver tissue showed that the protein level of CYP7A1 in male rats was significantly lower than that in females in both young and middle-aged rats, and it also showed that the protein level of SHP in male rats was significantly higher than that in females. In ileum tissue, the protein level of SHP in male rats was also significantly higher than that in females in both young and middle-aged rats. However, no similar phenomenon occurred in old rats (Figure 6). Furthermore, the mRNA levels of most genes related to the bile acid signaling pathway (especially *SHP*, *FXR*, *CYP7A1, and ASBT*) matched the levels of their corresponding proteins.

The above results suggested that there are significant sex differences in the mRNA and protein levels of the bile acid signaling pathway in SD rats, particularly *FXR*, *SHP*, *CYP7A1*, and *ASBT*.

### T regulated the expression of genes related to the bile acid signaling pathway

**Figure 7:**
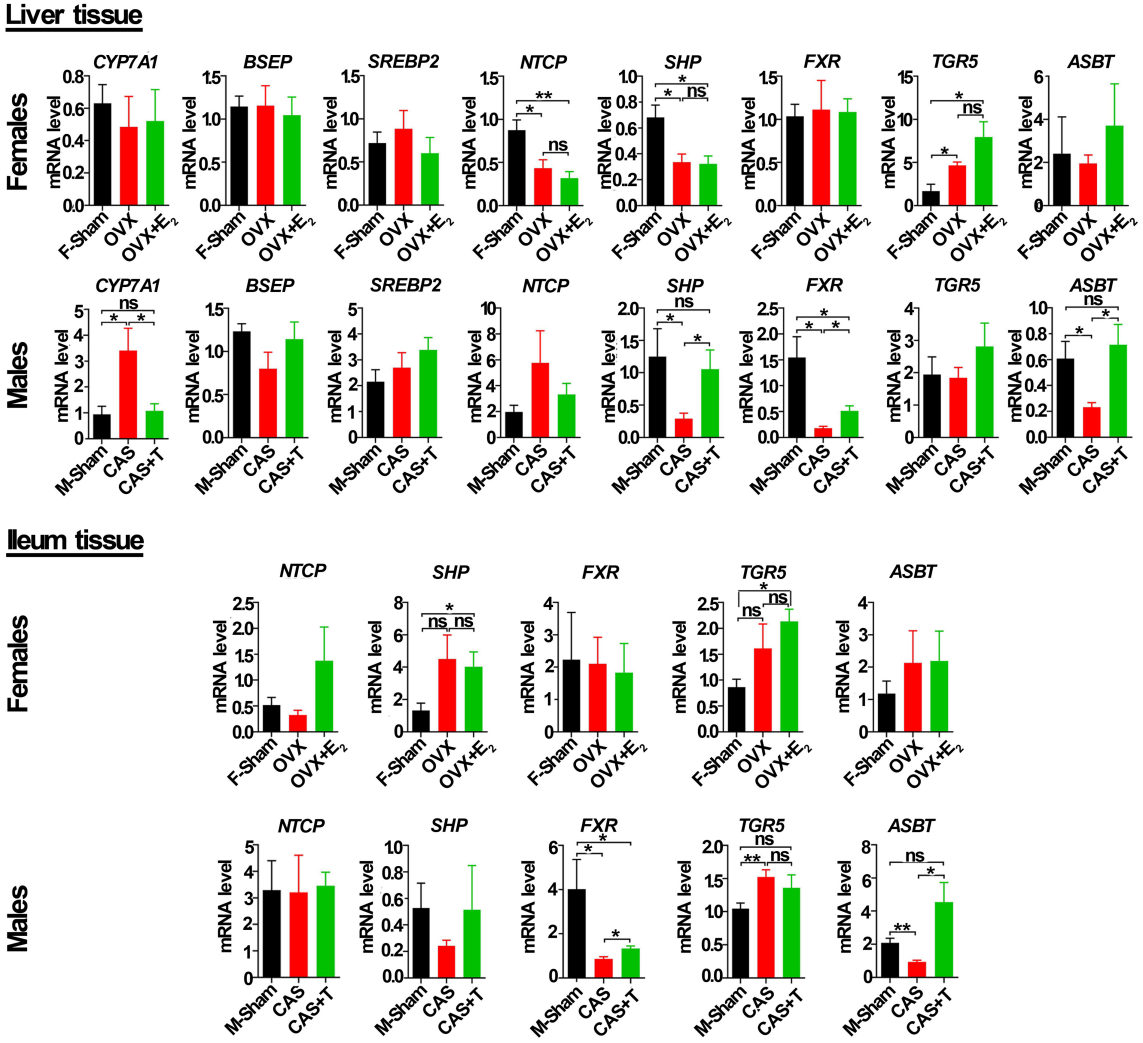
T regulated the mRNA level of genes in the bile acid signaling pathway in liver and ileum tissue from middle-aged rats (females and males). Individuals were grouped by Sham, castration (CAS), CAS + T, ovariectomy (OVX), and OVX + E2. *CYP7A1*, *BSEP*, *SREBP2*, *NTCP*, *SHP*, *FXR*, *TGR5* and *ASBT* relative mRNA abundances were determined by real-time PCR analysis, and relative gene pressions were normalized with *β-actin* and *GAPDH* (*n* = 5–6). F stands for females, and M stands for males. Values are presented as the mean ± SEM of three technical repetitions. Differences were assessed by an one-way ANOVA and denoted as follows: *p < 0.05; **p < 0.01; ***p < 0.001; ns *p* > 0.05. Three biological replications were performed and all results were similar.

To determine the basis for this variation in gene expression between the sexes related to the bile acid signaling pathway, we detected the mRNA and protein levels of these genes among the sham groups, GDX groups, and GDX + hormone groups in middle-aged rats. As shown in Figure 7, compared with the male sham group, the mRNA levels of *FXR*, *SHP,* and *ASBT* gene in liver tissue were significantly reduced in the castration (CAS) group, while the mRNA level of the *CYP7A1* gene was significantly increased in CAS group, and the expression of these genes was restored or partially restored following the addition of T to CAS group. Additionally, a significant decrease in the mRNA level of *ASBT* in the ileum was observed in the CAS group when compared to the male sham group. However, the inclusion of T in the CAS group led to a restoration of the *ASBT* gene expression. However, no similar phenomenon occurred among the female sham group, ovariectomy (OVX) group, and OVX + E2 group in either the liver tissue or ileum from rats.

**Figure 8:**
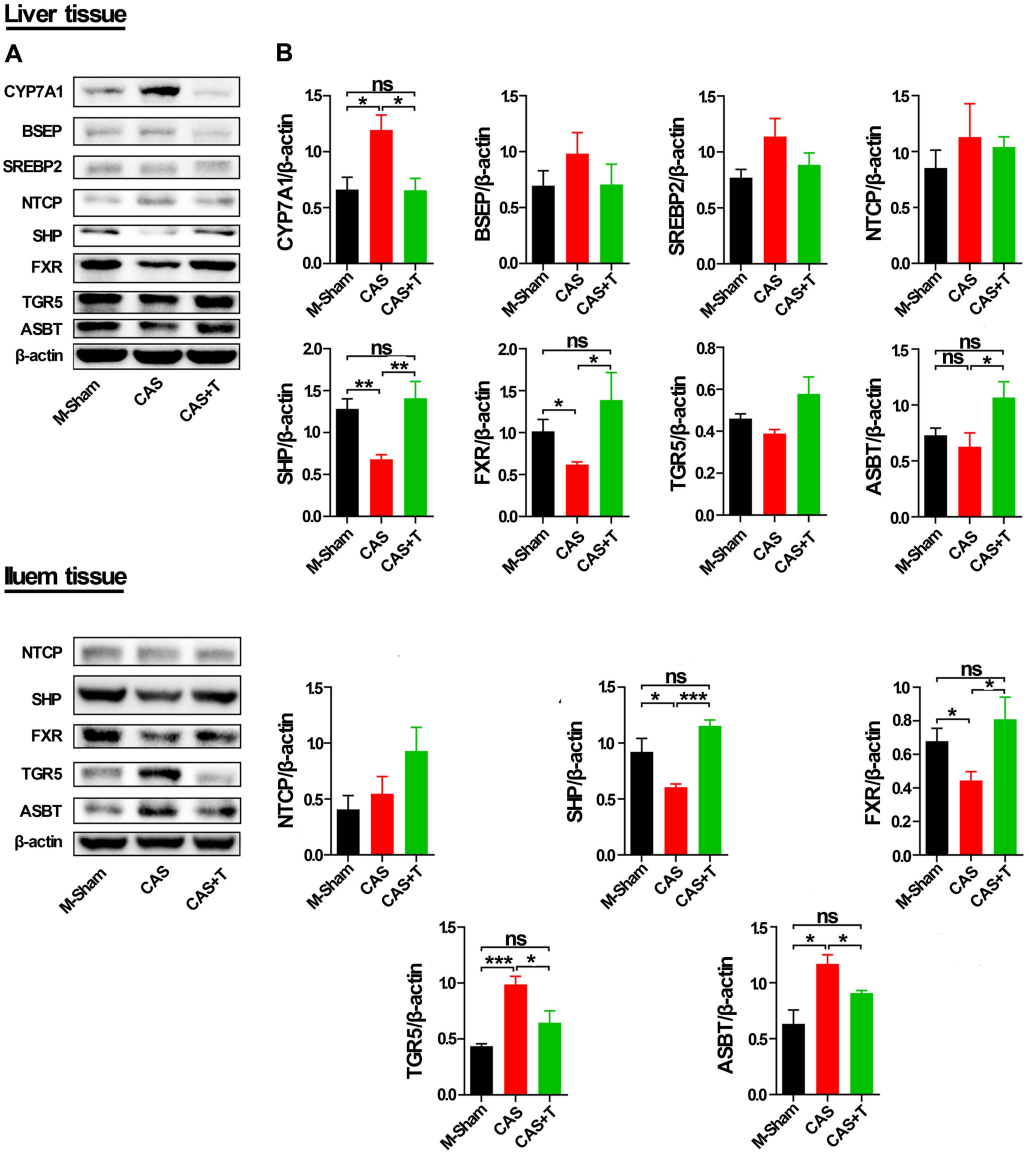
T regulated the expression of proteins in the bile acid signaling pathway in liver and ileum tissue from middle-aged male rats. Individuals were grouped by Sham, castration (CAS), CAS + T, ovariectomy (OVX) and OVX + E2. (A) CYP7A1, BSEP, SREBP2, NTCP, SHP, FXR, TGR5, and ASBT protein abundances were detected by western blot. (B) Quantification of CYP7A1, BSEP, SREBP2, NTCP, SHP, FXR, TGR5, and ASBT was normalized with β-actin (*n* = 5–6). Values are presented as the mean ± SEM of three technical repetitions. Differences were assessed by an one-way ANOVA and denoted as follows: *p < 0.05; **p < 0.01; ***p < 0.001; ns *p* > 0.05. Three biological replications were performed and all results were similar.

As shown by Western blots analysis (Figure 8; Supplementary Figure S7), compared with the male sham group, the protein levels of FXR and SHP in liver tissue were also significantly reduced in CAS group, while the protein level of the CYP7A1 was significantly increased in CAS group, and the protein expression of these genes was restored or partially restored following the addition of T to CAS group. Investigation into the protein level of ASBT in liver tissue revealed that, when comparing the male sham group with the CAS group, no appreciable difference was noted in the protein expression of ASBT. Yet, a notable upsurge in ASBT protein expression was observed in the CAS + T group, in contrast to the CAS group. Conversely, when juxtaposed with the male sham group, the CAS + T group did not exhibit a significant alteration in the ASBT protein level. Moreover, as compared with the male sham group, the protein levels of FXR and SHP in the ileum were significantly reduced in the CAS group, and the protein expression of these genes was restored following the addition of T to the CAS group. The protein levels of the TGR5 and ASBT were significantly increased in CAS and restored following the addition of T to CAS. However, no similar phenomenon occurred among the female sham group, OVX group, and OVX + E2 group in either the liver tissue or ileum from rats.

Furthermore, in immunofluorescent analysis of liver and ileum tissues, the protein level of SHP was significantly reduced in the CAS group as compared with the male sham group, and the expression of the protein was restored following the addition of T to CAS. Compared with the male sham group, the protein level of CYP7A1 was also significantly increased in CAS, and the protein expression of CYP7A1 was restored following the addition of T to CAS in liver tissue (Supplementary Figure S8).

**Figure 9:**
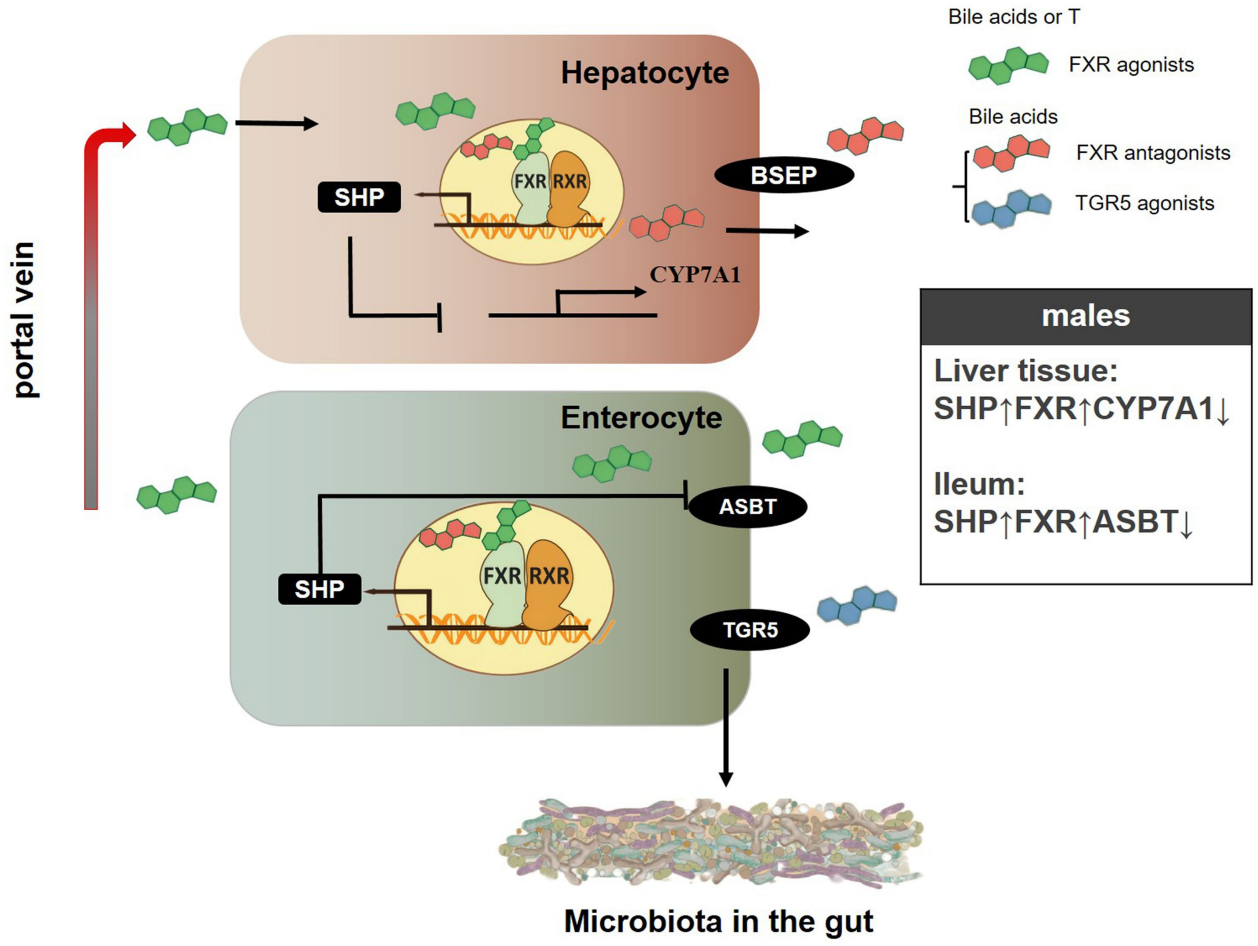
A schematic showing the possible mechanisms of gender-specific gut microbiota composition in SD rats. “↓” represents the decrease in protein levels of genes related to the bile acid signaling pathway, while “↑” represents the increase in protein levels of genes related to the bile acid signaling pathway.

In summary, the introduction of T enhanced the expression of genes such as *FXR*, *SHP*, and *ASBT* at both the mRNA and protein levels in liver tissue of SD rats, while concurrently decreasing the levels of *CYP7A1* mRNA and protein. Additionally, T treatment upregulated the protein expression of FXR and SHP in the ileum of SD rats and downregulated the protein levels of ASBT in the same region. The above results suggested that sex differences and T might affect the structure of the gut microbiota by regulating the mRNA and protein expression of genes related to the bile acid signaling pathway in SD rats (Figure 9).

## Discussion

### Structural differences of the intestinal flora in rats of different sexes

Gender dimorphism is a common feature of immune responses and numerous diseases, including inflammation, autoimmune diseases, infectious diseases, Parkinson’s disease, T2DM, cardiovascular diseases, Alzheimer’s disease, and cancer (Reckelhoff and Samson, 2015; Ortona et al., 2016; Dart, 2020; Di Florio et al., 2020; Gay et al., 2021; Kautzky-Willer et al., 2023; Dai et al., 2023; Patel and Kompoliti, 2023; Lopez-Lee et al., 2024; Puttur and Lloyd, 2024). An abundance of data demonstrates that the gut microbiota is involved in many of the diseases mentioned above. However, the mechanisms that mediate these connections are poorly understood, emphasizing the need to enhance our understanding of the genetic and environmental factors that affect microbial composition. 16S rRNA gene sequencing technology is an effective and commonly used method for detecting bacterial species and can be accurate to the genus level (James, 2018; Buetas et al., 2024). In this study, 16S rRNA gene sequencing technology was used to analyze structural differences in the intestinal microflora of male and female rats. In middle-aged rats, it was found that *Staphylococcus*, *Clostridiaceae*, and *Jeotgalicoccus* were enriched in male feces, while *Akkermansia*, *Lactobacillus*, and *Verrucomicrobiaceae* were enriched in female feces (Figure 1D). These microbes are closely related to multiple diseases. For example, *Staphylococcus* is responsible for human and animal infections globally, causing serious conditions such as bacteremia, wound infections, suppurative lesions, mastitis, and others (Stefani and Goglio, 2010; Contreras and Rodríguez, 2011; Rocha Balzan et al., 2023; Arumugam and Kielian, 2024; Westgeest et al., 2024). Significant effort and resources have been dedicated to researching and developing methods and medications to combat this bacterium (Chand et al., 2023; Dai et al., 2024). Several studies have shown that the lack or decreased abundance of *Akkermansia* is linked with immunologic function and multiple diseases (such as obesity, diabetes, liver steatosis, inflammation, and response to cancer immunotherapies; Cani et al., 2022; Jensen et al., 2024; Niu et al., 2024). Moreover, the bacterial community structure of fecal stools and jejunum of middle-aged and young rats of different sexes differed (Figure 2). Further, when the samples were taken from different parts of rats, the gut microbiota composition also differed (Figures 1, 2; Supplementary Figure S1), which may be caused by differences in the intestinal environment and other factors. The notion that we have uncovered sex-based disparities in gut microbiota is consistent with findings from previous research, adding weight to our conclusions (Org et al., 2016; Gan et al., 2023; Lledós et al., 2023; Le Bras, 2024; Ortiz-Alvarez de la Campa et al., 2024). Nevertheless, the evidence regarding the influence of gender on the gut microbiota’s composition has yielded mixed results. We speculated that the failure of some studies to find significant sex effects (Lay et al., 2005; Human Microbiome Project Consortium, 2012) could be attributed to the interference of variables like diet, age, host, and other factors (Costello et al., 2009; Kovacs et al., 2011; Carmody et al., 2015; Campaniello et al., 2022; Kawamoto et al., 2023; McCallum and Tropini, 2024). Indeed, we did not observe significant differences in the composition of the intestinal flora for both jejunum and stool samples or the level of serum T between sexes in old rats (Figures 2B,E,F,I). Org et al. (2016) also reported that many differences in composition appeared to be obscured by genetic background effects on the microbiota. Besides, other studies have also highlighted the significance of gender in the interplay between gut microbiota and environmental elements, such as dietary habits (Shastri et al., 2015; Bolnick et al., 2014; D'Archivio et al., 2022). In young rats, PCoA plots of Bray-Curtis distances of fecal and jejunal samples showed that no clear patterns differentiated fecal samples from males and females. In contrast, clear patterns differentiated jejunal samples from males and females (Figures 2C,D). The observed changes in gut microbiota composition could be attributed to multiple factors, including age, variations across different segments of the intestinal tract, and a range of influencing factors. Our prior study has also elucidated that the composition of the gut microbiota varies distinctly across different regions of the intestinal tract, and the effect of Traditional Chinese medicine (TCM) on the gut microbiota of rats had gender differences (Duan et al., 2022). Considering the profound interplay between the gut microbiome and the body’s immunity, alongside its implications for a spectrum of diseases including inflammation, metabolic diseases, cardiovascular diseases, tumors, and psychiatric disorders (Thaiss et al., 2016; Haneishi et al., 2023; Wang et al., 2023; Liu et al., 2023; Luqman et al., 2024; Pan et al., 2024; Roje et al., 2024), our research offers compelling insights into the gender-specific aspects of these conditions. This suggested that therapies aimed at modulating the gut microbiota might need to be tailored to each gender for optimal efficacy.

For gut microbiota test samples, it is important to note that in intestinal flora test samples, the jejunum is a significant part of the small intestine, while stool is excreted through the large intestine. The small intestine plays a crucial role in the digestion and absorption of macro and micronutrients, while the large intestine is essential for water absorption, facilitating adequate defecation, and housing intestinal microbiota (Ber et al., 2021). Our previous studies have demonstrated a significant disparity in the dominant flora between the large and small intestines (Duan et al., 2022; Zhang et al., 2023). Furthermore, research has indicated that the human jejunum possesses an indigenous microbiota distinct from that found in the oral cavity and colon (Sundin et al., 2017). Fecal samples are a non-invasive and sustainable method for observing animal gut microbiota. While fecal samples may not capture the real-time dynamic changes in bacterial populations in the animal gut, they reflect the overall gut microbiota composition (He et al., 2019). This allows for a better understanding of the structure and differences in animal gut microbiota. Therefore, to comprehensively reflect sex differences in gut microbiota, we selected the jejunum portion of the small intestine (along with its contents) and fecal samples excreted by the large intestine for analysis. Our previous study showed that TCM’s impacts on gut microbiota varied according to the intestinal segment and showed distinct quantitative and temporal regularity (Zhang et al., 2023). Here, we found significant differences in the composition of the microbiota between jejunum and feces (Supplementary Figure S1). The information provided implies that when developing pharmaceutical interventions aimed at modulating the gut microbiota, it is crucial to take into account the distinct segments of the intestine. This consideration ensures that drugs are effectively delivered to the intended sites and that their impact on the gut microbiota is appropriately managed across different regions of the gastrointestinal tract. In addition, for the sample size problem, a larger sample size is crucial for accurately capturing the variations in microbial composition among the groups, thereby enhancing the robustness of the data. Cost considerations and the imperative to ensure the continuity of the experiment led us to initially employ a study design involving 12 rats based on pertinent literature (Chen et al., 2018; Zhao et al., 2018; Yao et al., 2020; Gan et al., 2023), we acknowledge the potential factors leading to attrition, such as surgical interventions and inter-rat aggression during the feeding process.

### Relationship between the intestinal microflora structure and sex hormones in rats

Sex hormones play a crucial role in the development and balance of the human body, and changes in their levels can impact an individual’s internal stability (Rizzetto et al., 2018; Shin et al., 2019; Aref et al., 2024). Therefore, it is critical to maintain the body’s balance of sex hormones. In our study, except for older rats, the T levels in young and adult SD rats were significantly gender-specific, and the T levels in male rats were significantly higher than that in females, suggesting that T might affect the structure of the gut microbiota. However, E2 levels in male rats were also significantly higher than those in females, which may be due to hormone secretion related to age, and E2 levels in older female rats were significantly higher than those in males. Interestingly, the concentration of T in middle-aged rats was higher than that in both young and old rats (Figures 1F, 2G,I), which may be caused by all the physiological and pathological changes that damage body function accumulated during the aging process, which is consistent with the results reported by Decaroli and Rochira (2017). In previous studies, hormone replacement therapy (drugs that give patients exogenous hormone activity) was used to treat hormone deficiency or hypogonadism diseases (Mameli et al., 2023; Heidelbaugh and Belakovskiy, 2024). Estrogen is secreted primarily by the ovaries, and androgens are secreted primarily by the testicles. Following genital organ resection, the level of sex hormones in the body is unbalanced, and hormone replacement therapy is given to restore the balance of sex hormones. In the past few years, extensive research has also elucidated the pivotal role of sex hormones in the etiology and progression of various conditions, including tumors, cardiovascular issues, inflammatory responses, and metabolic disorders (Costa et al., 2020; Kumar et al., 2022; Hargrove-Wiley and Fingleton, 2023; Santos et al., 2023; Kan et al., 2024; Zhu D et al., 2024). Studies have found that in young and middle-aged people, the prevalence of type 2 diabetes is higher in men, but with age, the prevalence of women gradually increases and exceeds that of men (Kautzky-Willer et al., 2023). This may be related to hormonal changes in the body. Garcia-Fernandez et al. (2024) reported that a sex-specific dysbiosis in the intestinal microbiota is associated with coronary heart disease, potentially contributing to the observed sex disparity in the incidence of cardiovascular diseases. Bosch et al. (2024) found that sodium oligomeric mannitate has sex-specific effects on gut microbiota, reducing amyloidosis and reactive microglia in the brain. Therefore, it will be a trend to consider the involvement of sex hormones in the treatment of related diseases.

In our study, the testes of male rats were removed and the ovaries of female rats were removed, and then the rats underwent sex hormone therapy to observe the changes in the intestinal flora structure in each group. 16S rRNA gene sequencing demonstrated that the jejunum microflora diversity in the male surgery group was significantly reduced compared with that in the sham operation group (Figure 3G). The jejunum microflora in the male surgery group was similar to that in the sham operation group following T supplementation, and there was no significant difference in the Shannon index between the sham operation group and the male surgery group (Figure 3G). However, the PCoA analysis indicated that while the introduction of T post-gonadectomy seemed to mitigate the pronounced alterations typically associated with gonadectomy, there was still a distinct separation between the sham control group and CAS + T group on the composition of the gut microbiota (Figures 3B,H; Supplementary Tables S6, S7). The altered composition of the gut microbiota following gonadectomy may be influenced by factors beyond hormones alone, or it could be a result of the limited sample size we examined. These results indicated that T regulates the intestinal flora structure and increases the diversity of intestinal flora in male rats. In female rats, however, examining the female rat gut microbiota following gonadectomy, our analysis of *α*-diversity and PCoA uncovered notable distinctions. Notably, a clear delineation was observed between the sham control group and the GDX group. However, even after the introduction of E2 post-gonadectomy, a further distinct separation was still evident between the sham control group and the OVX + E2 group, highlighting persistent compositional differences in the gut microbiota (Figures 3A,G; Supplementary Figure S4; Supplementary Tables S6, S7), with the results confirming that sex hormones, particularly T, affect the intestinal microflora structure of rats. This finding is consistent with previous studies (Org et al., 2016).

Interestingly, sham control males exhibited more abundance of fecal *Staphylococcus* than GDX males, and the abundance of *Streptococcus* and *Romboutsia* were more abundant in sham controls as compared to GDX males in the jejunum, and again, the abundance of these microbes was restored or partially restored following the addition of T to CAS (Figures 3D,K,L). This is consistent with the phenomenon that the abundance of *Staphylococcus* in males was significantly higher than that in female rats in terms of fecal samples, and the abundance of *Streptococcus* and *Romboutsia* in males was significantly higher than that in female rats in terms of jejunal samples (Figure 1D). These microbes are closely related to multiple diseases. Previous research also has confirmed that *Romboutsia* was positively correlated with indicators of body weight (including waistline and body mass index) and serum lipids (including low-density lipoprotein, triglyceride, and total cholesterol; Zeng et al., 2019; Yin et al., 2023). In addition, T supplementation therapy was a longstanding treatment for hypogonadal men with metabolic diseases and atherosclerosis (Kumar et al., 2022; Sakamuri et al., 2024). Taken together, these data suggested that the gut microbiota may be involved in the regulation of sexual dimorphism of diseases.

### T regulated the expression of genes related to the bile acid signaling pathway and affected the gut microbiota structure

Numerous studies have shown that bile acid is a key factor in the regulation of the host intestinal microbial population (Wahlström et al., 2016; Quinn et al., 2020; Li et al., 2022; He et al., 2023). The conversion of cholesterol to bile acids occurs mainly through classical pathways in liver cells and alternative (acidic) pathways in tissues. This classical pathway is the main pathway of bile acid synthesis in the human body, with the bile acid synthesis catalyzed by this pathway accounting for 90% of the total bile in the human liver (Vlahcevic et al., 1991; Xiao et al., 2023). In the classical pathway, CYP7A1 is a key rate-limiting enzyme that catalyzes the conversion of cholesterol into primary bile acids, which plays an important role in maintaining the stable internal environment of cholesterol (Zhang et al., 2022; Yu Cai Lim and Kiat Ho, 2024). The Enterohepatic circulation is an important feature of bile acid metabolism; after eating, cholesterol in the liver is catalyzed by bile acid production of the classic rate-limiting enzyme CYP7A1. Bile acids are then secreted from the liver cells into the canaliculi and stored in the gallbladder. During lipid uptake, bile flows into the duodenum, which is subsequently absorbed in the terminal ileum and transported back to the liver via the portal vein (Houten and Auwerx, 2004; Ridlon and Gaskins, 2024). Most bile acids at the distal ileum are effectively reabsorbed by ASBT protein into intestinal cells and transported back to the liver (Park et al., 2022; Xiao et al., 2024). In hepatocytes, activation of FXR protein induces the expression of BSEP protein, which is responsible for the secretion of bile acids from hepatocytes into the ciliary ducts–an essential step for bile flow formation, enterohepatic circulation of bile salts, and lipid digestion (Chen et al., 2022; Zhao J. et al., 2023; Yao et al., 2024). Conversely, bile acid entry into hepatocytes is mainly accomplished by sodium-dependent NTCP transport (Park et al., 2022). The negative feedback of bile acid into the hepato-enteric circulation is mediated by SHP protein, which is a downstream target regulated by FXR protein. When activated by FXR, SHP can inhibit the expression of CYP7A1 and NTCP in liver cells. Moreover, SHP can promote the expression of FXR in enterocytes (Quilang et al., 2022; Liu C. et al., 2024; Yao et al., 2024).

In our investigation, the administration of T significantly amplified the expression of *FXR*, *SHP*, and *ASBT* genes at both the mRNA and protein levels within the liver tissue of middle-aged rats. Simultaneously, the expression of the *CYP7A1* gene was suppressed. Moreover, in the ileum of these same rats, T continued to augment the expression of *FXR* and *SHP* genes at both the mRNA and protein levels, while specifically inhibiting the expression of the *ASBT* gene at the protein level (Figures 7, 8; Supplementary Figure S8). These findings align with the observed gender-specific differences in the expression of these genes in male and female rats (Figures 4–6). This phenomenon may be because FXR can promote the expression of SHP and activation of FXR can inhibit the expression of CYP7A1. Jonker et al. determined that SHP can inhibit the expression of ASBT in enterocytes (Jonker et al., 2012). Interestingly, in the ileum, the mRNA level of *SHP* had no gender differences, but the protein level of SHP was higher in male rats compared to females in middle-aged rats. in middle-aged rats. In young rats, the mRNA level of *SHP* had no gender differences, but the protein level of SHP was higher in male rats compared to females in liver tissue. The expression of the *CYP7A1* gene also had a similar phenomenon in the ileum tissue of young rats. Notably, the mRNA level of *ASBT* was higher in male rats compared to females in liver tissues, but the protein level of ASBT had no gender differences in older rats (Figures 4, 6). Moreover, research conducted within the context of gonadectomy and hormone replacement therapy experiments revealed discordance between the mRNA and protein levels of the *ASBT* gene in the ileum tissue of middle-aged male rats (Figures 7, 8). The observed discrepancies between the mRNA level and protein level of *ASBT* in liver tissue from older rats, *SHP* in the ileum of middle-aged rats, and *CYP7A1* in the ileum of young rats, as well as *ASBT* in the ileum tissue of middle-aged male rats, could be attributed to the occurrence of post-transcriptional modifications. Additionally, in middle-aged rats in T promoted the expression of *ASBT* in liver tissue and inhibited the expression of *ASBT* in the ileum, and compared with the male sham groups, the mRNA and protein levels of the *TGR5* gene in the ileum were significantly increased in CAS, which was restored following the addition of T to CAS. This may be because bile acid promotes the expression of enterocyte *TGR5* when bile acid enters the intestine, and when too much intestinal bile acid is accumulated, the secretion of liver ASBT increases. This can allow bile acid to be reabsorbed from the intestine back to the liver to participate in hepatoenteric circulation. This mechanism ensures that these important compounds, which are essential for digestion and the absorption of dietary fats, are not lost but rather recycled and utilized efficiently by the body.

An imbalance in the gut microbiota and disruptions in bile acid metabolism interact in a detrimental cycle, potentially contributing to the development of metabolic disorders, cardiovascular issues, and cognitive decline (Mahmoudian Dehkordi et al., 2019; Wei et al., 2020; Chen B. et al., 2023; Lu et al., 2024). Fang et al. (2015) found that bile acids changed the composition of gut microbiota by activating FXR, reducing body weight, and improving insulin sensitivity. FXR agonists have been shown to prevent diseases such as cardiometabolic diseases, cholestasis, atherosclerosis, T2DM, obesity, and liver disease (Hambruch et al., 2012; Fang et al., 2015; Zhang et al., 2016; Li et al., 2020; Ghosh et al., 2023; Huang et al., 2024; Kim et al., 2024). When gut microbiota is disturbed, the ASBT-mediated reabsorption process of bile acid will be destroyed, and the expression of liver acetyl-CoA carboxylase 2 will be increased, thus aggravating the metabolic damage of the body (Agus et al., 2021). Secondary bile acids can effectively activate TGR5 signaling and induce the release of glucagon-like peptide-1, thereby improving glucose homeostasis (Liu R, et al., 2024; Yue et al., 2024).

Furthermore, whether it pertains to pharmaceuticals or groundbreaking research on the gut microbiota, exemplified by the study presented here, the ultimate aim is to drive forward the prevention and treatment of related diseases with unwavering determination and innovation. Traditional biological experiments often require time-consuming and labor-intensive approaches in their search for mechanisms of disease. However, in recent years, a plethora of cutting-edge models and techniques have revolutionized drug development and the treatment of associated diseases (Badkas et al., 2021; Yang et al., 2022; Chen Z.H. et al., 2023b; Qu et al., 2024). For instance, Zhao B.W. et al. (2023) integrated higher and lower-order biological information for drug repositioning using graph representation learning. Additionally, Zhao B.W. et al. (2024a) proposed a heterogeneous information network learning model with neighborhood-level structural representation for predicting lncRNA-miRNA interactions, which are closely related to the treatment of human diseases. Furthermore, Zhao B.W. et al. (2024b) introduced a novel motif-aware miRNA-disease association (MDA) prediction model, namely MotifMD, to predict the association between miRNAs and diseases. Moreover, the identification of proteins that interact with drug compounds has been recognized as an important aspect of the drug discovery process. *In silico* prediction of self-interacting proteins (SIPs) has become a crucial component of proteomics (Chen Z.H. et al., 2023b). Chen Z.H. et al. (2023b) proposed GraphCPIs: A novel graph-based computational model for potential compound-protein interactions. This computer-aided approach can identify high-quality compound-protein interaction candidates in real time. In conclusion, in future studies related to drugs, gut microbiota, and diseases, the signal pathway of drug regulation of gut microbiota and its interaction with proteins, small molecule RNAs, genes, and other related diseases will be predicted in advance through network-based computing methods and models. Subsequently, the relevant signal pathway and intermolecular interaction will be verified through biological experiments. This approach accurately and swiftly determines how drugs, gut microbiota, and disease interact, thereby facilitating research.

In summary, except for a few studies (Lay et al., 2005; Kovacs et al., 2011; Human Microbiome Project Consortium, 2012), most studies have found sex differences in gut microbiota (Org et al., 2016; Hansen et al., 2019; Shin et al., 2019; Gan et al., 2023, Santos-Marcos et al., 2023; Le Bras, 2024). Moreover, Org et al. (2016) also speculated that sex hormones interfere with the composition of intestinal flora. However, the exact mechanism remains unclear. Here, A study of SD rats reaffirmed the presence of sex differences in gut microbiota, particularly in middle-aged and young rats. This study compared the gut microbiota structure and levels of T and E2 in middle-aged, young, and old rats. The findings revealed that the gut microbiota structure of rats with sex differences in serum T levels also exhibited sex differences, and vice versa. Moreover, the intervention of sex hormones in the gut microbiota structure was confirmed through gonadectomy and hormone replacement therapy experiments. Finally, the study also compared the expression of genes and proteins associated with bile acid signaling pathways in the liver and ileum for the first time in middle-aged, elderly, young, gonadectomized, and hormone replacement therapy-treated rats. Our results provide one possible mechanism for sex differences in gut microbiota, shedding light on the strategic targeting of the “T-bile acid-gut microbiota” axis for disease prevention and management strategies.

## Conclusion

In young and adult SD rats, the composition of the gut microbiota, T levels, and mRNA and protein levels of genes related to the bile acid signaling pathway were significantly gender-specific. T levels in male rats were significantly higher than that in females. Moreover, gonadectomy and hormone replacement therapy indicated that the differences in the gut microbiota and mRNA and protein levels of genes related to the bile acid signaling pathway were mediated by T. These findings point to potential targets for disease prevention and management techniques by indicating that sex differences and T levels may alter the composition of the gut microbiota via the bile acid signaling pathway.

## Data Availability

The datasets presented in this study can be found in online repositories. The names of the repository/repositories and accession number(s) can be found in the article/Supplementary material.

## References

[ref1] Agus A. Clément K. Sokol H. (2021). Gut microbiota-derived metabolites as central regulators in metabolic disorders. Gut 70, 1174–1182. doi: 10.1136/gutjnl-2020-323071, PMID: 33272977 PMC8108286

[ref2] Alves C. J. de Santana L. P. dos Santos A. J. de Oliveira G. P. Duobles T. Scorisa J. M.. (2011). Early motor and electrophysiological changes in transgenic mouse model of amyotrophic lateral sclerosis and gender differences on clinical outcome. Brain Res. 1394, 90–104. doi: 10.1016/j.brainres.2011.02.060, PMID: 21354109

[ref3] Aref Y. Fat S. C. Ray E. (2024). Recent insights into the role of hormones during development and their functional regulation. Front. Endocrinol. 15:1340432. doi: 10.3389/fendo.2024.1340432 PMC10841574 38318293

[ref4] Arumugam P. Kielian T. (2024). Metabolism shapes immune responses to *staphylococcus aureus*. J. Innate Immun. 16, 12–30. doi: 10.1159/000535482, PMID: 38016430 PMC10766399

[ref5] Badkas A. De Landtsheer S. Sauter T. (2021). Topological network measures for drug repositioning. Brief. Bioinform. 22:bbaa357. doi: 10.1093/bib/bbaa357 33348366 PMC8294518

[ref6] Benedek G. Zhang J. Nguyen H. Kent G. Seifert H. A. Davin S.. (2017). Estrogen protection against EAE modulates the microbiota and mucosal-associated regulatory cells. J. Neuroimmunol. 310, 51–59. doi: 10.1016/j.jneuroim.2017.06.007, PMID: 28778445 PMC5570519

[ref7] Ber Y. García-Lopez S. Gargallo-Puyuelo C. J. Gomollón F. (2021). Small and large intestine (II): inflammatory bowel disease, short bowel syndrome, and malignant tumors of the digestive tract. Nutrients 13:2325. doi: 10.3390/nu13072325, PMID: 34371835 PMC8308711

[ref8] Bokulich N. A. Subramanian S. Faith J. J. Gevers D. Gordon J. I. Knight R.. (2013). Quality-filtering vastly improves diversity estimates from Illumina amplicon sequencing. Nat. Methods 10, 57–59. doi: 10.1038/nmeth.2276, PMID: 23202435 PMC3531572

[ref9] Bolnick D. I. Snowberg L. K. Hirsch P. E. Lauber C. L. Org E. Parks B.. (2014). Individual diet has sex-dependent effects on vertebrate gut microbiota. Nat. Commun. 5:4500. doi: 10.1038/ncomms5500 25072318 PMC4279269

[ref10] Bosch M. E. Dodiya H. B. Michalkiewicz J. Lee C. Shaik S. M. Weigle I. Q.. (2024). Sodium oligomannate alters gut microbiota, reduces cerebral amyloidosis and reactive microglia in a sex-specific manner. Mol. Neurodegener. 19:18. doi: 10.1186/s13024-023-00700-w 38365827 PMC10874048

[ref11] Brunkwall L. Orho-Melander M. (2017). The gut microbiome as a target for prevention and treatment of hyperglycaemia in type 2 diabetes: from current human evidence to future possibilities. Diabetologia 60, 943–951. doi: 10.1007/s00125-017-4278-3, PMID: 28434033 PMC5423958

[ref12] Buetas E. Jordán-López M. López-Roldán A. D'Auria G. Martínez-Priego L. De Marco G.. (2024). Full-length 16S rRNA gene sequencing by PacBio improves taxonomic resolution in human microbiome samples. BMC Genomics 25:310. doi: 10.1186/s12864-024-10213-5 38528457 PMC10964587

[ref13] Buffa J. A. Romano K. A. Copeland M. F. Cody D. B. Zhu W. Galvez R.. (2022). The microbial gbu gene cluster links cardiovascular disease risk associated with red meat consumption to microbiota L-carnitine catabolism. Nat. Microbiol. 7, 73–86. doi: 10.1038/s41564-021-01010-x, PMID: 34949826 PMC8732312

[ref14] Campaniello D. Corbo M. R. Sinigaglia M. Speranza B. Racioppo A. Altieri C.. (2022). How diet and physical activity modulate gut microbiota: evidence, and perspectives. Nutrients 14:2456. doi: 10.3390/nu14122456, PMID: 35745186 PMC9227967

[ref15] Cani P. D. Depommier C. Derrien M. Everard A. de Vos W. M. (2022). *Akkermansia muciniphila*: paradigm for next-generation beneficial microorganisms. Nat. Rev. Gastroenterol. Hepatol. 19, 625–637. doi: 10.1038/s41575-022-00631-9, PMID: 35641786

[ref16] Carmody R. N. Gerber G. K. Luevano J. M. Jr. Gatti D. M. Somes L. Svenson K. L.. (2015). Diet dominates host genotype in shaping the murine gut microbiota. Cell Host Microbe 17, 72–84. doi: 10.1016/j.chom.2014.11.010, PMID: 25532804 PMC4297240

[ref17] Chand U. Priyambada P. Kushawaha P. K. (2023). *Staphylococcus aureus* vaccine strategy: Promise and challenges. Microbiol. Res. 271:127362. doi: 10.1016/j.micres.2023.127362, PMID: 36958134

[ref18] Chávez-Talavera O. Tailleux A. Lefebvre P. Staels B. (2017). Bile acid control of metabolism and inflammation in obesity, type 2 diabetes, dyslipidemia, and nonalcoholic fatty liver disease. Gastroenterology 152, 1679–1694.e3. doi: 10.1053/j.gastro.2017.01.055, PMID: 28214524

[ref19] Chen B. Bai Y. Tong F. Yan J. Zhang R. Zhong Y.. (2023). Glycoursodeoxycholic acid regulates bile acids level and alters gut microbiota and glycolipid metabolism to attenuate diabetes. Gut Microbes 15:2192155. doi: 10.1080/19490976.2023.2192155 36967529 PMC10054359

[ref20] Chen M. Liao Z. Lu B. Wang M. Lin L. Zhang S.. (2018). Huang-Lian-Jie-Du-decoction ameliorates hyperglycemia and insulin resistant in association with gut microbiota modulation. Front. Microbiol. 9:2380. doi: 10.3389/fmicb.2018.02380 30349514 PMC6186778

[ref21] Chen H. Lu Y. Yang Y. Rao Y. (2023). A Drug Combination Prediction Framework Based on Graph Convolutional Network and Heterogeneous Information. IEEE/ACM Trans. Comput. Biol. Bioinform. 20, 1917–1925. doi: 10.1109/TCBB.2022.3224734, PMID: 36427284

[ref22] Chen Z. H. You Z. H. Zhang Q. H. Guo Z. H. Wang S. G. Wang Y. B. (2023a). In silico prediction methods of self-interacting proteins: an empirical and academic survey. Front. Comput. Sci. 17:173901. doi: 10.1007/s11704-022-1563-1

[ref23] Chen W. Zhang Q. Ding M. Yao J. Guo Y. Yan W.. (2022). Alcohol triggered bile acid disequilibrium by suppressing BSEP to sustain hepatocellular carcinoma progression. Chem. Biol. Interact. 356:109847. doi: 10.1016/j.cbi.2022.109847, PMID: 35149083

[ref24] Chen Z. H. Zhao B. W. Li J. Q. Guo Z. H. You Z. H. (2023b). GraphCPIs: A novel graph-based computational model for potential compound-protein interactions. Mol. Ther. Nucleic Acids. 32, 721–728. doi: 10.1016/j.omtn.2023.04.030, PMID: 37251691 PMC10209012

[ref25] Contreras G. A. Rodríguez J. M. (2011). Mastitis: comparative etiology and epidemiology. J. Mammary Gland Biol. Neoplasia 16, 339–356. doi: 10.1007/s10911-011-9234-0, PMID: 21947764

[ref26] Costa A. R. Lança de Oliveira M. Cruz I. Gonçalves I. Cascalheira J. F. Santos C. R. A. (2020). The sex bias of cancer. Trends Endocrinol. Metab. 31, 785–799. doi: 10.1016/j.tem.2020.07.002, PMID: 32900596

[ref27] Costello E. K. Lauber C. L. Hamady M. Fierer N. Gordon J. I. Knight R. (2009). Bacterial community variation in human body habitats across space and time. Science 326, 1694–1697. doi: 10.1126/science.1177486, PMID: 19892944 PMC3602444

[ref28] Dai M. Ouyang W. Yu Y. Wang T. Wang Y. Cen M.. (2024). IFP35 aggravates *Staphylococcus aureus* infection by promoting Nrf2-regulated ferroptosis. J. Adv. Res. 62, 143–154. doi: 10.1016/j.jare.2023.09.042, PMID: 37777065 PMC11331171

[ref29] Dai Z. Wang S. Guo X. Wang Y. Yin H. Tan J.. (2023). Gender dimorphism in hepatocarcinogenesis-DNA methylation modification regulated X-chromosome inactivation escape molecule XIST. Clin. Transl. Med. 13:e 1518. doi: 10.1002/ctm2.1518 PMC10751514 38148658

[ref30] D'Archivio M. Santangelo C. Silenzi A. Scazzocchio B. Varì R. Masella R. (2022). Dietary EVOO polyphenols and gut microbiota interaction: are there any sex/gender influences? Antioxidants. 11:1744. doi: 10.3390/antiox11091744, PMID: 36139818 PMC9495659

[ref31] Dart A. (2020). Sexual dimorphism in cancer. Nat. Rev. Cancer 20:627. doi: 10.1038/s41568-020-00304-2, PMID: 32918061

[ref32] Decaroli M. C. Rochira V. (2017). Aging and sex hormones in males. Virulence 8, 545–570. doi: 10.1080/21505594.2016.1259053, PMID: 27831823 PMC5538340

[ref33] Di Florio D. N. Sin J. Coronado M. J. Atwal P. S. Fairweather D. (2020). Sex differences in inflammation, redox biology, mitochondria and autoimmunity. Redox Biol. 31:101482. doi: 10.1016/j.redox.2020.101482, PMID: 32197947 PMC7212489

[ref34] Donaldson G. P. Ladinsky M. S. Yu K. B. Sanders J. G. Yoo B. B. Chou W. C.. (2018). Gut microbiota utilize immunoglobulin A for mucosal colonization. Science 360, 795–800. doi: 10.1126/science.aaq0926, PMID: 29724905 PMC5973787

[ref35] Down R. H. Whiting M. J. Watts J. M. Jones W. (1983). Effect of synthetic oestrogens and progestagens in oral contraceptives on bile lipid composition. Gut 24, 253–259. doi: 10.1136/gut.24.3.253, PMID: 6826111 PMC1419945

[ref36] Duan X. Xie X. Zhu C. Duan Z. Chen R. Xu J.. (2022). Sex difference of effect of *Sophora flavescens* on gut microbiota in rats. Evid. Based Complement. Alternat. Med. 2022:4552904. doi: 10.1155/2022/4552904, PMID: 35341152 PMC8941563

[ref37] Fang S. Suh J. M. Reilly S. M. (2015). Intestinal FXR agonism promotes adipose tissue browning and reduces obesity and insulin resistance. Nat. Med. 21, 159–165. doi: 10.1038/nm.3760, PMID: 25559344 PMC4320010

[ref38] Gan Y. Wu Y. J. Dong Y. Q. Li Q. Wu S. G. Jin Y. Q.. (2023). The study on the impact of sex on the structure of gut microbiota of bamboo rats in China. Front. Microbiol. 14:1276620. doi: 10.3389/fmicb.2023.1276620 38164398 PMC10757957

[ref39] Gao C. Hou L. (2023). Branched chain amino acids metabolism in heart failure. Front. Nutr. 10:1279066. doi: 10.3389/fnut.2023.1279066 38075219 PMC10699197

[ref40] Gao R. Meng X. Xue Y. Mao M. Liu Y. Tian X.. (2022). Bile acids-gut microbiota crosstalk contributes to the improvement of type 2 diabetes mellitus. Front. Pharmacol. 13:1027212. doi: 10.3389/fphar.2022.1027212 36386219 PMC9640995

[ref41] Garcia-Fernandez H. Arenas-de Larriva A. P. Lopez-Moreno J. Gutierrez-Mariscal F. M. Romero-Cabrera J. L. Molina-Abril H.. (2024). Sex-specific differences in intestinal microbiota associated with cardiovascular diseases. Biol. Sex Differ. 15:7. doi: 10.1186/s13293-024-00582-7 38243297 PMC10797902

[ref42] Gay L. Melenotte C. Lakbar I. Mezouar S. Devaux C. Raoult D.. (2021). Sexual dimorphism and gender in infectious diseases. Front. Immunol. 12:698121. doi: 10.3389/fimmu.2021.698121, PMID: 34367158 PMC8339590

[ref43] Ghosh S. Devereaux M. W. Anderson A. L. El Kasmi K. C. Sokol R. J. (2023). Stat3 role in the protective effect of FXR Agonist in parenteral nutrition-associated cholestasis. Hepatol. Commun. 7:e0056. doi: 10.1097/HC9.0000000000000056, PMID: 36848082 PMC9974070

[ref44] Goodrich J. K. Waters J. L. Poole A. C. Sutter J. L. Koren O. Blekhman R.. (2014). Human genetics shape the gut microbiome. Cell 159, 789–799. doi: 10.1016/j.cell.2014.09.053, PMID: 25417156 PMC4255478

[ref45] Hallen-Adams H. E. Suhr M. J. (2017). Fungi in the healthy human gastrointestinal tract. Virulence 8, 352–358. doi: 10.1080/21505594.2016.1247140 27736307 PMC5411236

[ref46] Hambruch E. Miyazaki-Anzai S. Hahn U. Matysik S. Boettcher A. Perović-Ottstadt S.. (2012). Synthetic farnesoid X receptor agonists induce high-density lipoprotein-mediated transhepatic cholesterol efflux in mice and monkeys and prevent atherosclerosis in cholesteryl ester transfer protein transgenic low-density lipoprotein receptor (−/−) mice. J. Pharmacol. Exp. Ther. 343, 556–567. doi: 10.1124/jpet.112.196519, PMID: 22918042 PMC11047796

[ref47] Haneishi Y. Furuya Y. Hasegawa M. Picarelli A. Rossi M. Miyamoto J. (2023). Inflammatory bowel diseases and gut microbiota. Int. J. Mol. Sci. 24:3817. doi: 10.3390/ijms24043817, PMID: 36835245 PMC9958622

[ref48] Hansen M. E. B. Rubel M. A. Bailey A. G. Ranciaro A. Thompson S. R. Campbell M. C.. (2019). Population structure of human gut bacteria in a diverse cohort from rural Tanzania and Botswana. Genome Biol. 20:16. doi: 10.1186/s13059-018-1616-9 30665461 PMC6341659

[ref49] Hargrove-Wiley E. Fingleton B. (2023). Sex Hormones in breast cancer immunity. Cancer Res. 83, 12–19. doi: 10.1158/0008-5472.CAN-22-1829, PMID: 36279153

[ref50] He X. Gao X. Hong Y. Zhong J. Li Y. Zhu W.. (2024). High fat diet and high sucrose intake divergently induce dysregulation of glucose homeostasis through distinct gut microbiota-derived bile acid metabolism in mice. J. Agric. Food Chem. 72, 230–244. doi: 10.1021/acs.jafc.3c02909, PMID: 38079533

[ref51] He M. Gao J. Wu J. Zhou Y. Fu H. Ke S.. (2019). Host gender and androgen levels regulate gut bacterial taxa in pigs leading to sex-biased serum metabolite profiles. Front. Microbiol. 10:1359. doi: 10.3389/fmicb.2019.01359 31275280 PMC6591444

[ref52] He S. Li L. Yao Y. Su J. Lei S. Zhang Y.. (2023). Bile acid and its bidirectional interactions with gut microbiota: a review. Crit. Rev. Microbiol. 1-18. doi: 10.1080/1040841X.2023.2262020 37766478

[ref53] Heidelbaugh J. J. Belakovskiy A. (2024). Testosterone replacement therapy for male hypogonadism. Aust. Fam. Physician 109, 543–549. 38905552

[ref54] Hokanson K. C. Hernández C. Deitzler G. E. Gaston J. E. David M. M. (2024). Sex shapes gut-microbiota-brain communication and disease. Trends Microbiol. 32, 151–161. doi: 10.1016/j.tim.2023.08.013 37813734

[ref55] Houten S. M. Auwerx J. (2004). The enterohepatic nuclear receptors are major regulators of the enterohepatic circulation of bile salts. Ann. Med. 36, 482–491. doi: 10.1080/07853890410018790 15513299

[ref56] Hrapkiewicz K. Colby L. Denison P. (1998). Clinical laboratory animal medicine, K. (Ames, IO: Iowa State University Press).

[ref57] Hu C. Yang M. (2024). Trends of serum 25 (OH) vitamin D and association with cardiovascular disease and all-cause mortality: from NHANES survey cycles 2001-2018. Front. Nutr. 11:1328136. doi: 10.3389/fnut.2024.1328136 38371503 PMC10869563

[ref58] Huang W. Cao Z. Wang W. Yang Z. Jiao S. Chen Y.. (2024). Discovery of LH10, a novel fexaramine-based FXR agonist for the treatment of liver disease. Bioorg. Chem. 143:107071. doi: 10.1016/j.bioorg.2023.107071, PMID: 38199141

[ref59] Human Microbiome Project Consortium (2012). Structure, function and diversity of the healthy human microbiome. Nature 486, 207–214. doi: 10.1038/nature11234, PMID: 22699609 PMC3564958

[ref60] James G. (2018). Universal bacterial identification by PCR and DNA sequencing of 16S r RNA gene. Dordrecht: Springer, 209–214.

[ref61] Jensen M. Stenfelt L. Ricci Hagman J. Pichler M. J. Weikum J. Nielsen T. S.. (2024). *Akkermansia muciniphila* exoglycosidases target extended blood group antigens to generate ABO-universal blood. Nat. Microbiol. 9, 1176–1188. doi: 10.1038/s41564-024-01663-4, PMID: 38684911

[ref62] Jonker J. W. Liddle C. Downes M. (2012). FXR and PXR: potential therapeutic targets in cholestasis. J. Steroid Biochem. Mol. Biol. 130, 147–158. doi: 10.1016/j.jsbmb.2011.06.012, PMID: 21801835 PMC4750880

[ref63] Kadyan S. Park G. Wang B. Nagpal R. (2023). Dietary fiber modulates gut microbiome and metabolome in a host sex-specific manner in a murine model of aging. Front. Mol. Biosci. 10:1182643. doi: 10.3389/fmolb.2023.1182643 37457834 PMC10345844

[ref64] Kan Y. Peng Y. L. Zhao Z. H. Dong S. T. Xu Y. X. Ma X. T.. (2024). The impact of female sex hormones on cardiovascular disease: from mechanisms to hormone therapy. J. Geriatr. Cardiol. 21, 669–681. doi: 10.26599/1671-5411.2024.06.003, PMID: 38973823 PMC11224657

[ref65] Kang Z. R. Jiang S. Han J. X. Gao Y. Xie Y. Chen J.. (2024). Deficiency of BCAT2-mediated branched-chain amino acid catabolism promotes colorectal cancer development. Biochim. Biophys. Acta Mol. basis Dis. 1870:166941. doi: 10.1016/j.bbadis.2023.166941, PMID: 37926361

[ref66] Kaska L. Sledzinski T. Chomiczewska A. Dettlaff-Pokora A. Swierczynski J. (2016). Improved glucose metabolism following bariatric surgery is associated with increased circulating bile acid concentrations and remodeling of the gut microbiome. World J. Gastroenterol. 22, 8698–8719. doi: 10.3748/wjg.v22.i39.8698, PMID: 27818587 PMC5075546

[ref67] Kautzky-Willer A. Leutner M. Harreiter J. (2023). Sex differences in type 2 diabetes. Diabetologia 66, 986–1002. doi: 10.1007/s00125-023-05891-x, PMID: 36897358 PMC10163139

[ref68] Kawamoto S. Uemura K. Hori N. Takayasu L. Konishi Y. Katoh K.. (2023). Bacterial induction of B cell senescence promotes age-related changes in the gut microbiota. Nat. Cell Biol. 25, 865–876. doi: 10.1038/s41556-023-01145-5, PMID: 37169880

[ref69] Kim C. Tsai T. H. Lopez R. McCullough A. Kasumov T. (2024). Obeticholic acid's effect on HDL function in MASH varies by diabetic status. Lipids. doi: 10.1002/lipd.12408 PMC11560728 39014264

[ref70] Kolodziejczyk A. A. Zheng D. Elinav E. (2019). Diet-microbiota interactions and personalized nutrition. Nat. Rev. Microbiol. 17, 742–753. doi: 10.1038/s41579-019-0256-8, PMID: 31541197

[ref71] Kovacs A. Ben-Jacob N. Tayem H. Halperin E. Iraqi F. A. Gophna U. (2011). Genotype is a stronger determinant than sex of the mouse gut microbiota. Microb. Ecol. 61, 423–428. doi: 10.1007/s00248-010-9787-2, PMID: 21181142

[ref72] Kumar S. Khatri M. Memon R. A. Velastegui J. L. Podaneva K. Z. Gutierrez D. B.. (2022). Effects of testosterone therapy in adult males with hypogonadism and T2DM: A meta-analysis and systematic review. Diabetes Metab. Syndr. 16:102588. doi: 10.1016/j.dsx.2022.102588 35952509

[ref73] Lay C. Rigottier-Gois L. Holmstrøm K. Rajilic M. Vaughan E. E. de Vos W. M.. (2005). Colonic microbiota signatures across five northern European countries. Appl. Environ. Microbiol. 71, 4153–4155. doi: 10.1128/AEM.71.7.4153-4155.2005, PMID: 16000838 PMC1169042

[ref74] Le Bras A. (2024). Sex differences in microbiota. Lab. Anim. 53:218. doi: 10.1038/s41684-024-01430-2, PMID: 39341532

[ref75] Li T. T. Chen X. Huo D. Arifuzzaman M. Qiao S. Jin W. B.. (2024). Microbiota metabolism of intestinal amino acids impacts host nutrient homeostasis and physiology. Cell Host Microbe 32, 661–675.e10. doi: 10.1016/j.chom.2024.04.004, PMID: 38657606 PMC11636940

[ref76] Li M. Wang S. Li Y. Zhao M. Kuang J. Liang D.. (2022). Gut microbiota-bile acid crosstalk contributes to the rebound weight gain after calorie restriction in mice. Nat. Commun. 13:2060. doi: 10.1038/s41467-022-29589-7 35440584 PMC9018700

[ref77] Li C. Yang J. Wang Y. Qi Y. Yang W. Li Y. (2020). Farnesoid X receptor agonists as therapeutic target for cardiometabolic diseases. Front. Pharmacol. 11:1247. doi: 10.3389/fphar.2020.01247 32982723 PMC7479173

[ref78] Liu C. Cai T. Cheng Y. Bai J. Li M. Gu B.. (2024). Postbiotics prepared using *lactobacillus reuteri* ameliorates ethanol-induced liver injury by regulating the FXR/SHP/SREBP-1c axis. Mol. Nutr. Food Res. 68:e2300927. doi: 10.1002/mnfr.202300927, PMID: 38937862

[ref79] Liu L. Wang H. Chen X. Zhang Y. Zhang H. Xie P. (2023). Gut microbiota and its metabolites in depression: from pathogenesis to treatment. EBioMedicine 90:104527. doi: 10.1016/j.ebiom.2023.104527, PMID: 36963238 PMC10051028

[ref80] Liu R. Wang J. Zhao Y. Zhou Q. Yang X. Gao Y.. (2024). Study on the mechanism of modified Gegen Qinlian decoction in regulating the intestinal flora-bile acid-TGR5 axis for the treatment of type 2 diabetes mellitus based on macro genome sequencing and targeted metabonomics integration. Phytomedicine 132:155329. doi: 10.1016/j.phymed.2023.155329, PMID: 38853123

[ref81] Lledós M. Prats-Sánchez L. Llucià-Carol L. Cárcel-Márquez J. Muiño E. Cullell N.. (2023). Ischaemic stroke patients present sex differences in gut microbiota. Eur. J. Neurol. 30, 3497–3506. doi: 10.1111/ene.15931, PMID: 37329328

[ref82] Lopez-Lee C. Torres E. R. S. Carling G. Gan L. (2024). Mechanisms of sex differences in Alzheimer's disease. Neuron 112, 1208–1221. doi: 10.1016/j.neuron.2024.01.024, PMID: 38402606 PMC11076015

[ref83] Lu Q. Chen J. Jiang L. Geng T. Tian S. Liao Y.. (2024). Gut microbiota-derived secondary bile acids, bile acids receptor polymorphisms, and risk of cardiovascular disease in individuals with newly diagnosed type 2 diabetes: a cohort study. Am. J. Clin. Nutr. 119, 324–332. doi: 10.1016/j.ajcnut.2023.08.023, PMID: 38309826

[ref84] Luqman A. Hassan A. Ullah M. Naseem S. Ullah M. Zhang L.. (2024). Role of the intestinal microbiome and its therapeutic intervention in cardiovascular disorder. Front. Immunol. 15:1321395. doi: 10.3389/fimmu.2024.1321395 38343539 PMC10853344

[ref85] Mahmoudian Dehkordi S. Arnold M. Nho K. Ahmad S. Jia W. Xie G.. (2019). Altered bile acid profile associates with cognitive impairment in Alzheimer's disease-An emerging role for gut microbiome. Alzheimers Dement. 15, 76–92. doi: 10.1016/j.jalz.2018.07.217 30337151 PMC6487485

[ref86] Mameli C. Orso M. Calcaterra V. Wasniewska M. G. Aversa T. Granato S.. (2023). Efficacy, safety, quality of life, adherence and cost-effectiveness of long-acting growth hormone replacement therapy compared to daily growth hormone in children with growth hormone deficiency: A systematic review and meta-analysis. Pharmacol. Res. 193:106805. doi: 10.1016/j.phrs.2023.106805, PMID: 37236413

[ref87] Markle J. G. Frank D. N. Mortin-Toth S. Robertson C. E. Feazel L. M. Rolle-Kampczyk U.. (2013). Sex differences in the gut microbiome drive hormone-dependent regulation of autoimmunity. Science 339, 1084–1088. doi: 10.1126/science.1233521, PMID: 23328391

[ref88] McCallum G. Tropini C. (2024). The gut microbiota and its biogeography. Nat. Rev. Microbiol. 22, 105–118. doi: 10.1038/s41579-023-00969-0, PMID: 37740073

[ref89] McDonald D. Price M. N. Goodrich J. Nawrocki E. P. DeSantis T. Z. Probst A.. (2012). An improved greengenes taxonomy with explicit ranks for ecological and evolutionary analyses of bacteria and archaea. ISME J. 6, 610–618. doi: 10.1038/ismej.2011.139, PMID: 22134646 PMC3280142

[ref90] Niu H. Zhou M. Zogona D. Xing Z. Wu T. Chen R.. (2024). *Akkermansia muciniphila*: a potential candidate for ameliorating metabolic diseases. Front. Immunol. 15:1370658. doi: 10.3389/fimmu.2024.1370658 38571945 PMC10987721

[ref91] Org E. Mehrabian M. Parks B. W. Shipkova P. Liu X. Drake T. A.. (2016). Sex differences and hormonal effects on gut microbiota composition in mice. Gut Microbes 7, 313–322. doi: 10.1080/19490976.2016.1203502, PMID: 27355107 PMC4988450

[ref92] Org E. Parks B. W. Joo J. W. Emert B. Schwartzman W. Kang E. Y.. (2015). Genetic and environmental control of host-gut microbiota interactions. Genome Res. 25, 1558–1569. doi: 10.1101/gr.194118.115, PMID: 26260972 PMC4579341

[ref93] Ortiz-Alvarez de la Campa M. Curtis-Joseph N. Beekman C. Belenky P. (2024). Gut biogeography accentuates sex-related differences in the murine microbiome. Microorganisms 12:221. doi: 10.3390/microorganisms12010221, PMID: 38276206 PMC10821414

[ref94] Ortona E. Pierdominici M. Maselli A. Veroni C. Aloisi F. Shoenfeld Y. (2016). Sex-based differences in autoimmune diseases. Ann. Ist. Super. Sanita 52, 205–212. doi: 10.4415/ANN_16_02_12, PMID: 27364395

[ref95] Pan Y. Bu T. Deng X. Jia J. Yuan G. (2024). Gut microbiota and type 2 diabetes mellitus: a focus on the gut-brain axis. Endocrine 84, 1–15. doi: 10.1007/s12020-023-03640-z, PMID: 38227168

[ref96] Park J. H. Iwamoto M. Yun J. H. Uchikubo-Kamo T. Son D. Jin Z.. (2022). Structural insights into the HBV receptor and bile acid transporter NTCP. Nature 606, 1027–1031. doi: 10.1038/s41586-022-04857-0, PMID: 35580630 PMC9242859

[ref97] Patel R. Kompoliti K. (2023). Sex and gender differences in Parkinson's disease. Neurol. Clin. 41, 371–379. doi: 10.1016/j.ncl.2022.12.001, PMID: 37030964

[ref98] Pfaffl M. W. (2001). A new mathematical model for relative quantification in real-time RT-PCR. Nucleic Acids Res. 29:e45. doi: 10.1093/nar/29.9.e45, PMID: 11328886 PMC55695

[ref99] Puttur F. Lloyd C. M. (2024). Sex differences in tissue immunity. Science 384, 159–160. doi: 10.1126/science.ado8542, PMID: 38574173

[ref100] Qin J. Li R. Raes J. Arumugam M. Burgdorf K. S. Manichanh C.. (2010). A human gut microbial gene catalogue established by metagenomic sequencing. Nature 464, 59–65. doi: 10.1038/nature08821, PMID: 20203603 PMC3779803

[ref101] Qu X. Du G. Hu J. Cai Y. (2024). Graph-DTI: A new model for drug-target interaction prediction based on heterogenous network graph embedding. Curr. Comput. Aided Drug Des. 20, 1013–1024. doi: 10.2174/1573409919666230713142255, PMID: 37448360

[ref102] Qu W. Liu S. Zhang W. Zhu H. Tao Q. Wang H.. (2019). Impact of traditional Chinese medicine treatment on chronic unpredictable mild stress-induced depression-like behaviors: intestinal microbiota and gut microbiome function. Food Funct. 10, 5886–5897. doi: 10.1039/c9fo00399a 31464319

[ref103] Quilang R. C. Lui S. Forbes K. (2022). miR-514a-3p: a novel SHP-2 regulatory miRNA that modulates human cytotrophoblast proliferation. J. Mol. Endocrinol. 68, 99–110. doi: 10.1530/JME-21-0175, PMID: 34792485 PMC8789026

[ref104] Quinn R. A. Melnik A. V. Vrbanac A. Fu T. Patras K. A. Christy M. P.. (2020). Global chemical effects of the microbiome include new bile-acid conjugations. Nature 579, 123–129. doi: 10.1038/s41586-020-2047-9, PMID: 32103176 PMC7252668

[ref105] Razavi A. C. Potts K. S. Kelly T. N. Bazzano L. A. (2019). Sex, gut microbiome, and cardiovascular disease risk. Biol. Sex Differ. 10:29. doi: 10.1186/s13293-019-0240-z 31182162 PMC6558780

[ref106] Reckelhoff J. F. Samson W. K. (2015). Sex and gender differences in cardiovascular, renal and metabolic diseases. Am. J. Phys. Regul. Integr. Comp. Phys. 309, R1057–R1059. doi: 10.1152/ajpregu.00417.2015, PMID: 26447212 PMC4666959

[ref107] Renke G. Starling-Soares B. Baesso T. Petronio R. Aguiar D. Paes R. (2023). Effects of vitamin D on cardiovascular risk and oxidative stress. Nutrients 15:769. doi: 10.3390/nu15030769, PMID: 36771474 PMC9920542

[ref108] Ridlon J. M. Gaskins H. R. (2024). Another renaissance for bile acid gastrointestinal microbiology. Nat. Rev. Gastroenterol. Hepatol. 21, 348–364. doi: 10.1038/s41575-024-00896-2, PMID: 38383804 PMC11558780

[ref109] Rio P. Caldarelli M. Chiantore M. Ocarino F. Candelli M. Gasbarrini A.. (2024). Immune cells, gut microbiota, and vaccines: A gender perspective. Cells 13:526. doi: 10.3390/cells13060526, PMID: 38534370 PMC10969451

[ref110] Rizzetto L. Fava F. Tuohy K. M. Selmi C. (2018). Connecting the immune system, systemic chronic inflammation and the gut microbiome: The role of sex. J. Autoimmun. 92, 12–34. doi: 10.1016/j.jaut.2018.05.008, PMID: 29861127

[ref111] Rocha Balzan L. D. L. Rossato A. M. Riche C. V. W. Cantarelli V. V. D'Azevedo P. A. Valério de Lima A.. (2023). Staphylococcus argenteus Infections. Brazil. Microbiol. Spectr. 11:e0117922. doi: 10.1128/spectrum.01179-22, PMID: 36688721 PMC9927369

[ref112] Roje B. Zhang B. Mastrorilli E. Kovačić A. Sušak L. Ljubenkov I.. (2024). Gut microbiota carcinogen metabolism causes distal tissue tumours. Nature 632, 1137–1144. doi: 10.1038/s41586-024-07754-w, PMID: 39085612 PMC11358042

[ref113] Rothhammer V. Borucki D. M. Tjon E. C. Takenaka M. C. Chao C. C. Ardura-Fabregat A.. (2018). Microglial control of astrocytes in response to microbial metabolites. Nature 557, 724–728. doi: 10.1038/s41586-018-0119-x, PMID: 29769726 PMC6422159

[ref114] Rudolph K. Schneider D. Fichtel C. Daniel R. Heistermann M. Kappeler P. M. (2022). Drivers of gut microbiome variation within and between groups of a wild Malagasy primate. Microbiome. 10:28. doi: 10.1186/s40168-021-01223-6 35139921 PMC8827170

[ref115] Sakamuri A. Visniauskas B. Kilanowski-Doroh I. McNally A. B. Imulinde A. Kamau A.. (2024). Testosterone deficiency promotes arterial stiffening independent of sex chromosome complement. Biol. Sex Differ. 15:46. doi: 10.1186/s13293-024-00624-0 38845040 PMC11155160

[ref116] Santos J. D. Oliveira-Neto J. T. Tostes R. C. (2023). The cardiovascular subtleties of testosterone on gender-affirming hormone therapy. Am. J. Physiol. Heart Circ. Physiol. 325, H30–H53. doi: 10.1152/ajpheart.00015.2023, PMID: 37145958

[ref117] Santos-Marcos J. A. Mora-Ortiz M. Tena-Sempere M. Lopez-Miranda J. Camargo A. (2023). Interaction between gut microbiota and sex hormones and their relation to sexual dimorphism in metabolic diseases. Biol. Sex Differ. 14:4. doi: 10.1186/s13293-023-00490-2 36750874 PMC9903633

[ref118] Schnorr S. L. Candela M. Rampelli S. Centanni M. Consolandi C. Basaglia G.. (2014). Gut microbiome of the Hadza hunter-gatherers. Nat. Commun. 5:3654. doi: 10.1038/ncomms4654 24736369 PMC3996546

[ref119] Shamjana U. Vasu D. A. Hembrom P. S. Nayak K. Grace T. (2024). The role of insect gut microbiota in host fitness, detoxification and nutrient supplementation. Antonie Van Leeuwenhoek 117:71. doi: 10.1007/s10482-024-01970-0 38668783

[ref120] Shastri P. McCarville J. Kalmokoff M. Brooks S. P. Green-Johnson J. M. (2015). Sex differences in gut fermentation and immune parameters in rats fed an oligofructose-supplemented diet. Biol. Sex Differ. 6:13. doi: 10.1186/s13293-015-0031-0 26251695 PMC4527341

[ref121] Shin J. H. Park Y. H. Sim M. Kim S. A. Joung H. Shin D. M. (2019). Serum level of sex steroid hormone is associated with diversity and profiles of human gut microbiome. Res. Microbiol. 170, 192–201. doi: 10.1016/j.resmic.2019.03.003, PMID: 30940469

[ref122] Solon-Biet S. M. Cogger V. C. Pulpitel T. Wahl D. Clark X. Bagley E.. (2019). Branched chain amino acids impact health and lifespan indirectly via amino acid balance and appetite control. Nat. Metab. 1, 532–545. doi: 10.1038/s42255-019-0059-2, PMID: 31656947 PMC6814438

[ref123] Stefani S. Goglio A. (2010). Methicillin-resistant *Staphylococcus aureus*: related infections and antibiotic resistance. Int. J. Infect. Dis. 14, S19–S22. doi: 10.1016/j.ijid.2010.05.009, PMID: 20843722

[ref124] Sundin O. H. Mendoza-Ladd A. Zeng M. Diaz-Arévalo D. Morales E. Fagan B. M.. (2017). The human jejunum has an endogenous microbiota that differs from those in the oral cavity and colon. BMC Microbiol. 17:160. doi: 10.1186/s12866-017-1059-6 28716079 PMC5513040

[ref125] Takagi T. Naito Y. Inoue R. Kashiwagi S. Uchiyama K. Mizushima K.. (2019). Differences in gut microbiota associated with age, sex, and stool consistency in healthy Japanese subjects. J. Gastroenterol. 54, 53–63. doi: 10.1007/s00535-018-1488-5, PMID: 29926167

[ref126] Thaiss C. A. Zmora N. Levy M. Elinav E. (2016). The microbiome and innate immunity. Nature 535, 65–74. doi: 10.1038/nature18847, PMID: 27383981

[ref127] Tian F. Huang S. Xu W. Chen L. Su J. Ni H.. (2022). Compound K attenuates hyperglycemia by enhancing glucagon-like peptide-1 secretion through activating TGR5 via the remodeling of gut microbiota and bile acid metabolism. J. Ginseng Res. 46, 780–789. doi: 10.1016/j.jgr.2022.03.006, PMID: 36312739 PMC9597441

[ref128] Van der Giessen J. van der Woude C. J. Peppelenbosch M. P. Fuhler G. M. (2019). A Direct Effect of Sex Hormones on Epithelial Barrier Function in Inflammatory Bowel Disease Models. Cells 8:261. doi: 10.3390/cells8030261, PMID: 30893871 PMC6468635

[ref129] Vich Vila A. Collij V. Sanna S. Sinha T. Imhann F. Bourgonje A. R.. (2020). Impact of commonly used drugs on the composition and metabolic function of the gut microbiota. Nat. Commun. 11:362. doi: 10.1038/s41467-019-14177-z 31953381 PMC6969170

[ref130] Vlahcevic Z. R. Heuman D. M. Hylemon P. B. (1991). Regulation of bile acid synthesis. Hepatology 13, 590–600. doi: 10.1002/hep.1840130331, PMID: 1847897

[ref131] Wahlström A. Sayin S. I. Marschall H. U. Bäckhed F. (2016). Intestinal Crosstalk between Bile Acids and Microbiota and Its Impact on Host Metabolism. Cell Metab. 24, 41–50. doi: 10.1016/j.cmet.2016.05.005, PMID: 27320064

[ref132] Wang J. Gao Y. Ren S. Li J. Chen S. Feng J.. (2024a). Gut microbiota-derived trimethylamine N-Oxide: a novel target for the treatment of preeclampsia. Gut Microbes 16:2311888. doi: 10.1080/19490976.2024.2311888 38351748 PMC10868535

[ref133] Wang J. He M. Yang M. Ai X. (2024b). Gut microbiota as a key regulator of intestinal mucosal immunity. Life Sci. 345:122612. doi: 10.1016/j.lfs.2024.122612, PMID: 38588949

[ref134] Wang Z. Klipfell E. Bennett B. J. Koeth R. Levison B. S. Dugar B.. (2011). Gut flora metabolism of phosphatidylcholine promotes cardiovascular disease. Nature 472, 57–63. doi: 10.1038/nature09922, PMID: 21475195 PMC3086762

[ref135] Wang M. Ou Y. Yuan X. L. Zhu X. F. Niu B. Kang Z.. (2024). Heterogeneously elevated branched-chain/aromatic amino acids among new-onset type-2 diabetes mellitus patients are potentially skewed diabetes predictors. World J. Diabetes 15, 53–71. doi: 10.4239/wjd.v15.i1.53, PMID: 38313852 PMC10835491

[ref136] Wang J. Zhu N. Su X. Gao Y. Yang R. (2023). Gut-microbiota-derived metabolites maintain gut and systemic immune homeostasis. Cells 12:793. doi: 10.3390/cells12050793, PMID: 36899929 PMC10000530

[ref137] Wei M. Huang F. Zhao L. (2020). A dysregulated bile acid-gut microbiota axis contributes to obesity susceptibility. EBioMedicine 55:102766. doi: 10.1016/j.ebiom.2020.102766, PMID: 32408110 PMC7225614

[ref138] Westergaard D. Moseley P. Sørup F. K. H. Baldi P. Brunak S. (2019). Population-wide analysis of differences in disease progression patterns in men and women. Nat. Commun. 10:666. doi: 10.1038/s41467-019-08475-9 30737381 PMC6368599

[ref139] Westgeest A. C. Lambregts M. M. C. Fowler V. G. Jr. (2024). Reply to robertson: true *Staphylococcus aureus* bacteremia. Clin. Infect. Dis. 79, 568–569. doi: 10.1093/cid/ciad753, PMID: 38060808 PMC11327782

[ref140] Wong C. C. Yu J. (2023). Gut microbiota in colorectal cancer development and therapy. Nat. Rev. Clin. Oncol. 20, 429–452. doi: 10.1038/s41571-023-00766-x, PMID: 37169888

[ref141] Wu J. Shen H. Lv Y. He J. Xie X. Xu Z.. (2024). Age over sex: evaluating gut microbiota differences in healthy Chinese populations. Front. Microbiol. 15:1412991. doi: 10.3389/fmicb.2024.1412991 38974029 PMC11224521

[ref142] Wu X. M. Tan R. X. (2019). Interaction between gut microbiota and ethnomedicine constituents. Nat. Prod. Rep. 36, 788–809. doi: 10.1039/c8np00041g 30534698

[ref143] Xiao J. Dong L. W. Liu S. Meng F. H. Xie C. Lu X. Y.. (2023). Bile acids-mediated intracellular cholesterol transport promotes intestinal cholesterol absorption and NPC1L1 recycling. Nat. Commun. 14:6469. doi: 10.1038/s41467-023-42179-5 37833289 PMC10575946

[ref144] Xiao L. Estellé J. Kiilerich P. Ramayo-Caldas Y. Xia Z. Feng Q.. (2016). A reference gene catalogue of the pig gut microbiome. Nat. Microbiol. 1:16161. doi: 10.1038/nmicrobiol.2016.161 27643971

[ref145] Xiao Y. Jia Y. Q. Liu W. J. Niu C. Mai Z. H. Dong J. Q.. (2024). Pulsatilla decoction alleviates DSS-induced UC by activating FXR-ASBT pathways to ameliorate disordered bile acids homeostasis. Front. Pharmacol. 15:1399829. doi: 10.3389/fphar.2024.1399829 38974033 PMC11224520

[ref146] Xie G. Wang X. Zhao A. Yan J. Chen W. Jiang R.. (2017). Sex-dependent effects on gut microbiota regulate hepatic carcinogenic outcomes. Sci. Rep. 7:45232. doi: 10.1038/srep45232 28345673 PMC5366919

[ref147] Yang Y. Gao D. Xie X. Qin J. Li J. Lin H.. (2022). DeepIDC: A prediction framework of injectable drug combination based on heterogeneous information and deep Learning. Clin. Pharmacokinet. 61, 1749–1759. doi: 10.1007/s40262-022-01180-9, PMID: 36369328

[ref148] Yao Z. Chen L. Hu M. Meng F. Chen M. Wang G. (2024). The discovery of a new potent FXR agonist based on natural product screening. Bioorg. Chem. 143:106979. doi: 10.1016/j.bioorg.2023.106979, PMID: 37995646

[ref149] Yao Y. Yan L. Chen H. Wu N. Wang W. Wang D. (2020). Cyclocarya paliurus polysaccharides alleviate type 2 diabetic symptoms by modulating gut microbiota and short-chain fatty acids. Phytomedicine 77:153268. doi: 10.1016/j.phymed.2020.153268, PMID: 32663709

[ref150] Yin H. Huang J. Guo X. Xia J. Hu M. (2023). Romboutsia lituseburensis JCM1404 supplementation ameliorated endothelial function via gut microbiota modulation and lipid metabolisms alterations in obese rats. FEMS Microbiol. Lett. 370:fnad016. doi: 10.1093/femsle/fnad016 36869802

[ref151] Yu Cai Lim M. Kiat Ho H. (2024). Pharmacological modulation of cholesterol 7α-hydroxylase (CYP7A1) as a therapeutic strategy for hypercholesterolemia. Biochem. Pharmacol. 220:115985. doi: 10.1016/j.bcp.2023.115985, PMID: 38154545

[ref152] Yu L. Pan J. Guo M. Duan H. Zhang H. Narbad A.. (2024). Gut microbiota and anti-aging: Focusing on spermidine. Crit. Rev. Food Sci. Nutr. 64: 10419–10437. doi: 10.1080/10408398.2023.2224867 [Epubh ahead of print]., PMID: 37326367

[ref153] Yue Z. Zhao F. Guo Y. Zhang Y. Chen Y. He L.. (2024). *Lactobacillus reuteri* JCM 1112 ameliorates chronic acrylamide-induced glucose metabolism disorder via the bile acid-TGR5-GLP-1 axis and modulates intestinal oxidative stress in mice. Food Funct. 15, 6450–6458. doi: 10.1039/D4FO01061B, PMID: 38804210

[ref154] Zeng Q. Li D. He Y. Li Y. Yang Z. Zhao X.. (2019). Discrepant gut microbiota markers for the classification of obesity-related metabolic abnormalities. Sci. Rep. 9:13424. doi: 10.1038/s41598-019-49462-w 31530820 PMC6748942

[ref155] Zhang Q. Duan X. Chen R. Duan Z. Zhu C. Yu Q.. (2023). Effects of Cortex meliae on the intestinal flora in rats. J. Biobased Mater. Bio. 17, 742–750. doi: 10.1166/jbmb.2023.2323

[ref156] Zhang Y. Lou J. W. Kang A. Zhang Q. Zhou S. K. Bao B. H.. (2020). Kansuiphorin C and Kansuinin A ameliorate malignant ascites by modulating gut microbiota and related metabolic functions. J. Ethnopharmacol. 249:112423. doi: 10.1016/j.jep.2019.112423, PMID: 31765764

[ref157] Zhang J. Hou Y. Zhang Z. Shi Y. Wang Z. Song G. (2024). Correlation between serum vitamin E and HOMA-IR in patients with T2DM. Diabetes Metab. Syndr. Obes. 17, 1833–1843. doi: 10.2147/DMSO.S450738 38680996 PMC11055560

[ref158] Zhang J. Wang X. Jiang H. Yang F. Du Y. Wang L.. (2022). MicroRNA-185 modulates CYP7A1 mediated cholesterol-bile acid metabolism through post-transcriptional and post-translational regulation of FoxO1. Atherosclerosis 348, 56–67. doi: 10.1016/j.atherosclerosis.2022.03.007, PMID: 35287950

[ref159] Zhang H. M. Wang X. Wu Z. H. Liu H. L. Chen W. Zhang Z. Z.. (2016). Beneficial effect of farnesoid X receptor activation on metabolism in a diabetic rat model. Mol. Med. Rep. 13, 2135–2142. doi: 10.3892/mmr.2016.4761, PMID: 26782298

[ref160] Zhao B. W. He Y. Z. Su X. R. Yang Y. Li G. D. Huang Y. A.. (2024b). Motif-aware miRNA-disease association prediction via hierarchical attention network. IEEE J. Biomed. Health Inform. 28, 4281–4294. doi: 10.1109/JBHI.2024.3383591, PMID: 38557614

[ref161] Zhao J. Song G. Weng F. Li Y. Zou B. Jin J.. (2023). The choleretic role of tauroursodeoxycholic acid exacerbates alpha-naphthylisothiocyanate induced cholestatic liver injury through the FXR/BSEP pathway. J. Appl. Toxicol. 43, 1095–1103. doi: 10.1002/jat.4446, PMID: 36787806

[ref162] Zhao B. W. Su S. R. Yang Y. Li D. X. Li G. D. Hu P. W.. (2024a). A heterogeneous information network learning model with neighborhood-level structural representation for predicting lncRNA-miRNA interactions. Comput. Struct. Biotechnol. J. 23, 2924–2933. doi: 10.1016/j.csbj.2024.06.032 PMC11832017 39963422

[ref163] Zhao B. W. Wang L. Hu P. W. Wong L. Su X. R. Wang B. Q.. (2023). Fusing higher and lower-order biological information for drug repositioning via graph representation learning. IEEE T. Emerg. Top. Com. 12, 1–14. doi: 10.1109/TETC.2023.3239949

[ref164] Zhao L. Zhang F. Ding X. Wu G. Lam Y. Y. Wang X.. (2018). Gut bacteria selectively promoted by dietary fibers alleviate type 2 diabetes. Science 359, 1151–1156. doi: 10.1126/science.aao5774, PMID: 29590046

[ref165] Zhu X. Chen Z. Zhang B. Xie S. Wang M. (2024). Bile acid injection regulated blood glucose in T2DM rats via the TGR5/GLP-1 rather than FXR/FGF15 pathway. Altern. Ther. Health Med. [Epub ahead of print]., PMID: 39038352

[ref166] Zhu D. Du Y. Zhu L. Alahmadi T. A. Hussein-Al-Ali S. H. Wang Q. (2024). Testosterone with silymarin improves diabetes-obesity comorbidity complications by modulating inflammatory responses and CYP7A1/ACC gene expressions in rats. Comb. Chem. High Throughput Screen. 27, 1999–2012. doi: 10.2174/0113862073272401231108054024, PMID: 37957854

