# Supplementary material for: Sex differences and testosterone interfere with the structure of the gut microbiota through the bile acid signaling pathway
Source: Front Microbiol. 2024 Oct 18;15:1421608. doi: 10.3389/fmicb.2024.1421608 (PMC11527610; doi:10.3389/fmicb.2024.1421608)
Supplement: Supplementary file 1 [file Data_Sheet_1.doc]

**Supplementary data**

**Table S1. Analysis of similarity (ANOSIM) of gut microbiota composition in middle-aged rats.**

| Breeding | Groups | R | *P*.value | Dissimilarity | Permutation |
| --- | --- | --- | --- | --- | --- |
| SD rats | Females-F VS Males-F | 0.18 | 0.02 | Bray-curtis | 999 |
|  | Females-J VS Males-J | 0.38 | 0.01 | Bray-curtis | 999 |
|  | Females-F VS Females-J | 0.70 | 1.00x10^-3^ | Bray-curtis | 999 |
|  | Males-F VS Males-J | 0.82 | 1.00x10^-3^ | Bray-curtis | 999 |

F stands for fecal samples; J stands for jejunal samples, n = 10 per group

**Table S2 Permutational multivariate analysis of variance (PERMANOVA) of gut microbiota composition in middle-aged rats.**

| Breeding | Groups | Df | SumsOfSqs | F.Model | R2 | *P*.value |
| --- | --- | --- | --- | --- | --- | --- |
| SD rats | Females-F VS Males-F | 1 | 0.26 | 2.17 | 0.11 | 0.02 |
|  | Females-J VS Males-J | 1 | 1.26 | 5.70 | 0.24 | 5.00x10^-3^ |
|  | Females-F VS Females-J | 1 | 2.26 | 14.13 | 0.44 | 1.00x10^-3^ |
|  | Males-F VS Males-J | 1 | 1.95 | 10.59 | 0.37 | 1.00x10^-3^ |

F stands for fecal samples; J stands for jejunal samples, n = 10 per group.

**Table S3. The influence of sex on the structure of intestinal microflora**

* *p* value were adjusted using Benjamini-Hochberg FDR

| Breeding Samples Taxonomy  (Genus) | | | Relative abundance  (%, Mean values±SD, n = 10) | | *p*-value  (adjusted)^*^ |
| --- | --- | --- | --- | --- | --- |
|  |  |  | Females Males | |  |
| SD rats | Feces | *Lactobacillus* | **10.85±5.74** | 3.61±2.81 | 7.25x10^-4^ |
|  |  | *Akkermansia* | **1.94±2.50** | 0.02±0.03 | 3.60x10^-3^ |
|  | Jejunum | *Prevotella* | **21.92+15.63** | 0.69±1.15 | 1.05x10^-3^ |
|  |  | *Roseburia* | **3.23±2.53** | 0.08±0.14 | 5.20x10^-3^ |
|  |  | *Romboutsia* | 4.11±2.80 | **11.48±10.53** | 0.03 |
|  |  | *Saccharibacteria_genera_incertae_sedis* | **1.70±1.15** | 0.42±0.51 | 0.01 |
|  |  | *Streptococcus* | 0.32±0.50 | **1.35±1.82** | 0.02 |
|  |  | *Oscilibacter* | **2.19±1.72** | 0.46±1.30 | 0.03 |

* *p* value were adjusted using Benjamini-Hochberg FDR

**Table S4. ANOSIM of gut microbiota composition in young and old** rats

| Classify | Groups | R | *P*.value | Dissimilarity | Permutation |
| --- | --- | --- | --- | --- | --- |
| Young | Females-F VS Males-F | 0.09 | 0.08 | Bray-curtis | 999 |
|  | Females-J VS Males-J | 0.29 | 6.00x10^-3^ | Bray-curtis | 999 |
|  | Females-F VS Females-J | 1.00 | 1.00x10^-3^ | Bray-curtis | 999 |
|  | Males-F VS Males-J | 0.99 | 1.00x10^-3^ | Bray-curtis | 999 |
| Old | Females-F VS Males-F | 0.14 | 0.07 | Bray-curtis | 999 |
|  | Females-J VS Males-J | 0.10 | 0.06 | Bray-curtis | 999 |
|  | Females-F VS Females-J | 1.00 | 1.00x10^-3^ | Bray-curtis | 999 |
|  | Males-F VS Males-J | 0.99 | 1.00x10^-3^ | Bray-curtis | 999 |

F stands for fecal samples; J stands for jejunal samples, n = 9–10 per group.

**Table S5. PERMANOVA of gut microbiota composition in young and old rats**

| Classify | | Groups | | Df | | SumsOfSqs | | F.Model | | R2 | | *P*.value | |
| --- | --- | --- | --- | --- | --- | --- | --- | --- | --- | --- | --- | --- | --- |
| Young | | Females-F VS Males-F | | 1 | | 0.07 | | 1.31 | | 0.07 | | 0.25 | |
|  | Females-J VS Males-J | | 1 | | 0.77 | | 3.46 | | 0.16 | | 9.00x10^-3^ | |  |
|  | Females-F VS Females-J | | 1 | | 2.46 | | 14.6 | | 0.48 | | 1.00x10^-3^ | |  |
|  | Males-F VS Males-J | | 1 | | 2.51 | | 14.8 | | 0.49 | | 1.00x10^-3^ | |  |
| Old | | Females-F VS Males-F | | 1 | | 0.29 | | 1.50 | | 0.09 | | 0.13 | |
|  | Females-J VS Males-J | | 1 | | 0.28 | | 1.92 | | 0.11 | | 0.09 | |  |
|  | | Females-F VS Females-J | | 1 | | 2.72 | | 16.2 | | 0.50 | | 1.00x10^-3^ | |
|  | | Males-F VS Males-J | | 1 | | 2.79 | | 16.2 | | 0.50 | | 1.00x10^-3^ | |

F stands for fecal samples; J stands for jejunal samples, n = 9–10 per group.


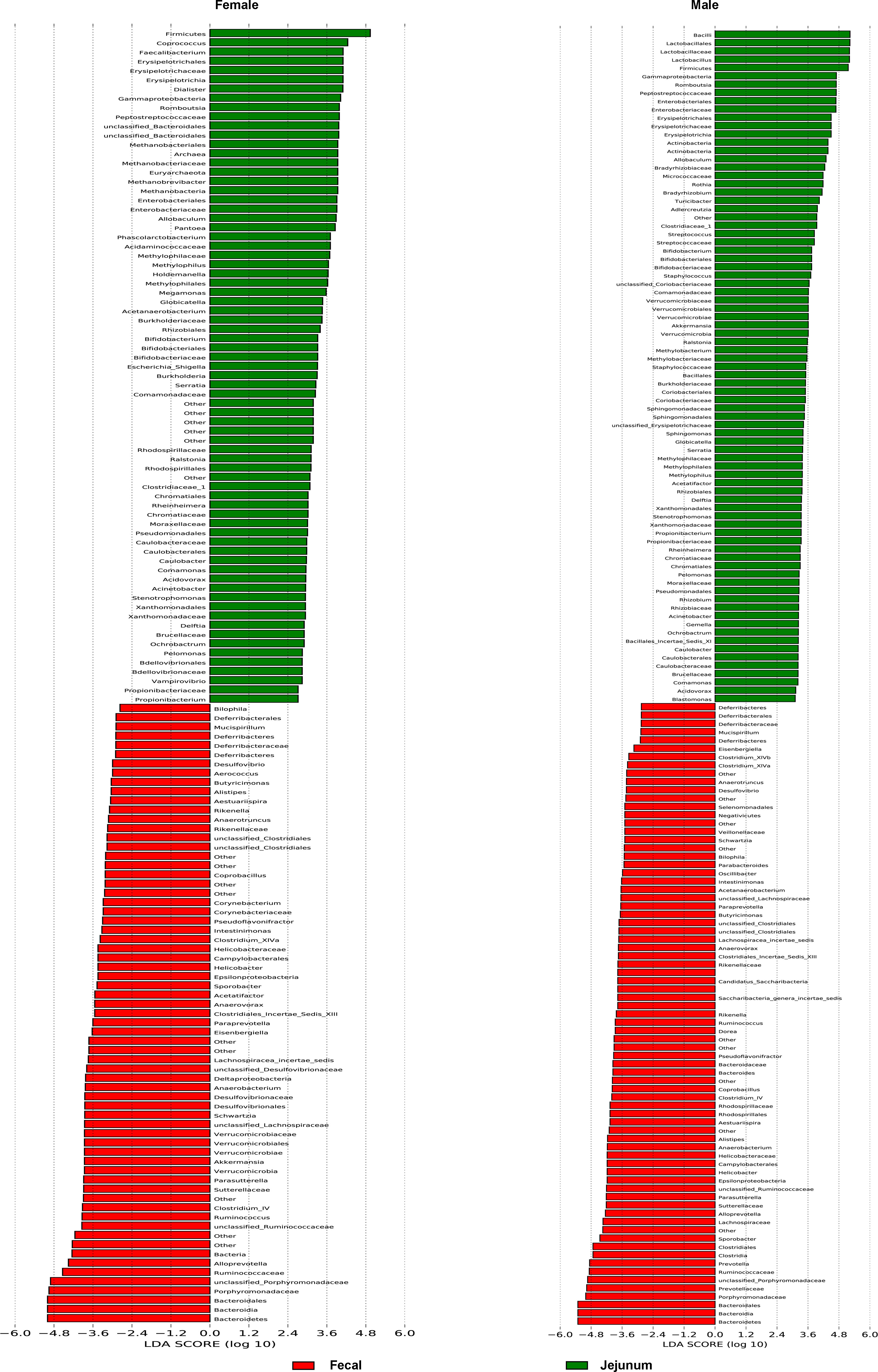


**Figure S1. Linear discriminant analysis (LDA) of gut microbiota composition between** [**faeces**](javascript:;) **and jejunum in middle-aged rats**. The LDA coupled with effect size measurements identified the most differentially abundant genus level taxa between fecal and jejunal samples from rats rats. all taxa detected at the phylum to genus level. n = 10 per group.

**
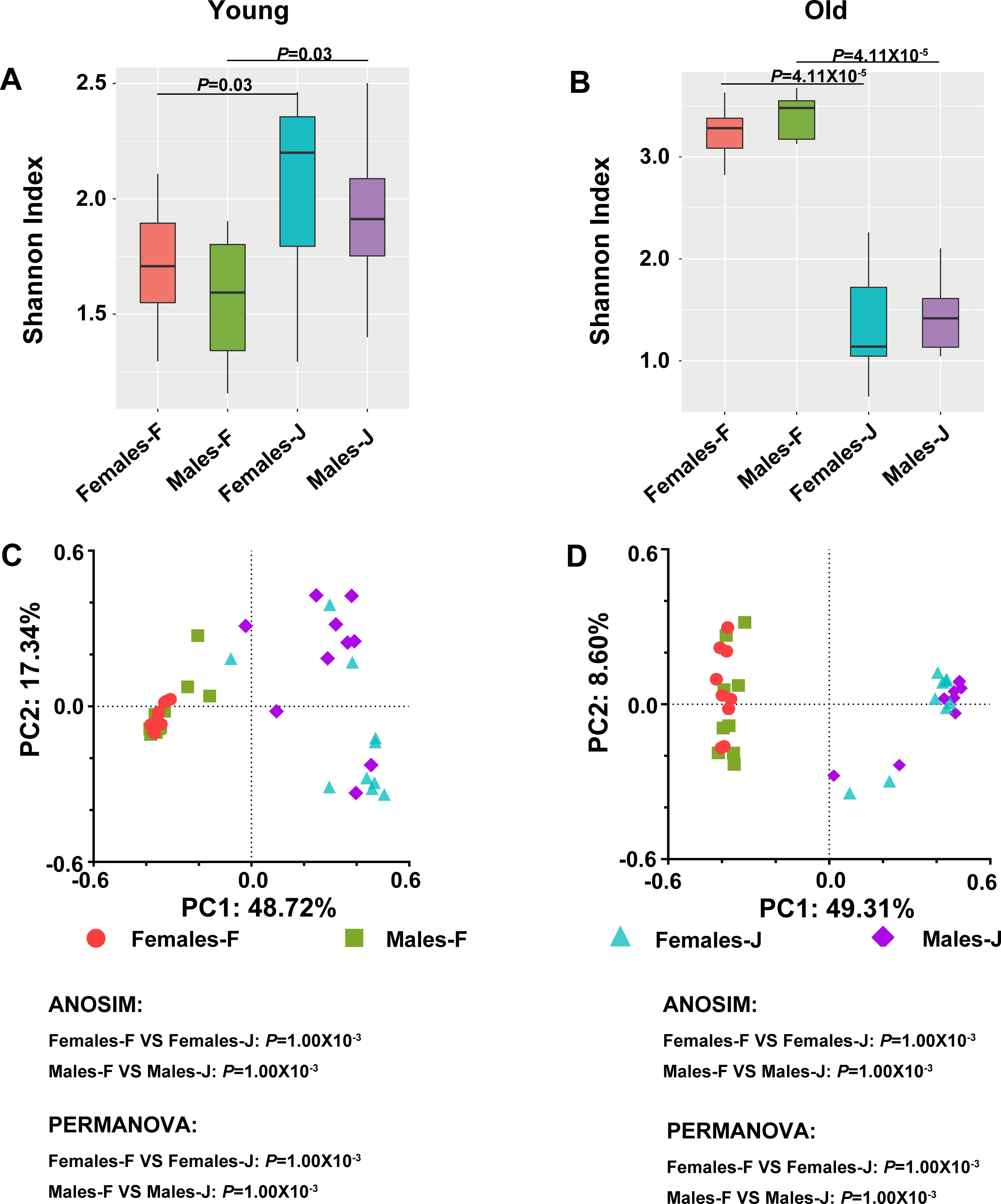
**

**Figure S2. Spatial specificity gut microbiota composition in young and old rats.** (A-B) within group mean α diversity, *P* values from Mann–Whitney U test. (C-D) Principal coordinate analysis (PCoA) plot of Bray-Curtis distances，analysis of similarity (ANOSIM) and permutational multivariate analysis of variance (PERMANOVA) of the microbial communities between fecal and jejunal samples. *P* values from ANOSIM and PERMANOVA are shown for sex. In this experiment, rats rats purchased Hunan. n = 9–10 per group, F stands for fecal samples; J stands for jejunal samples.


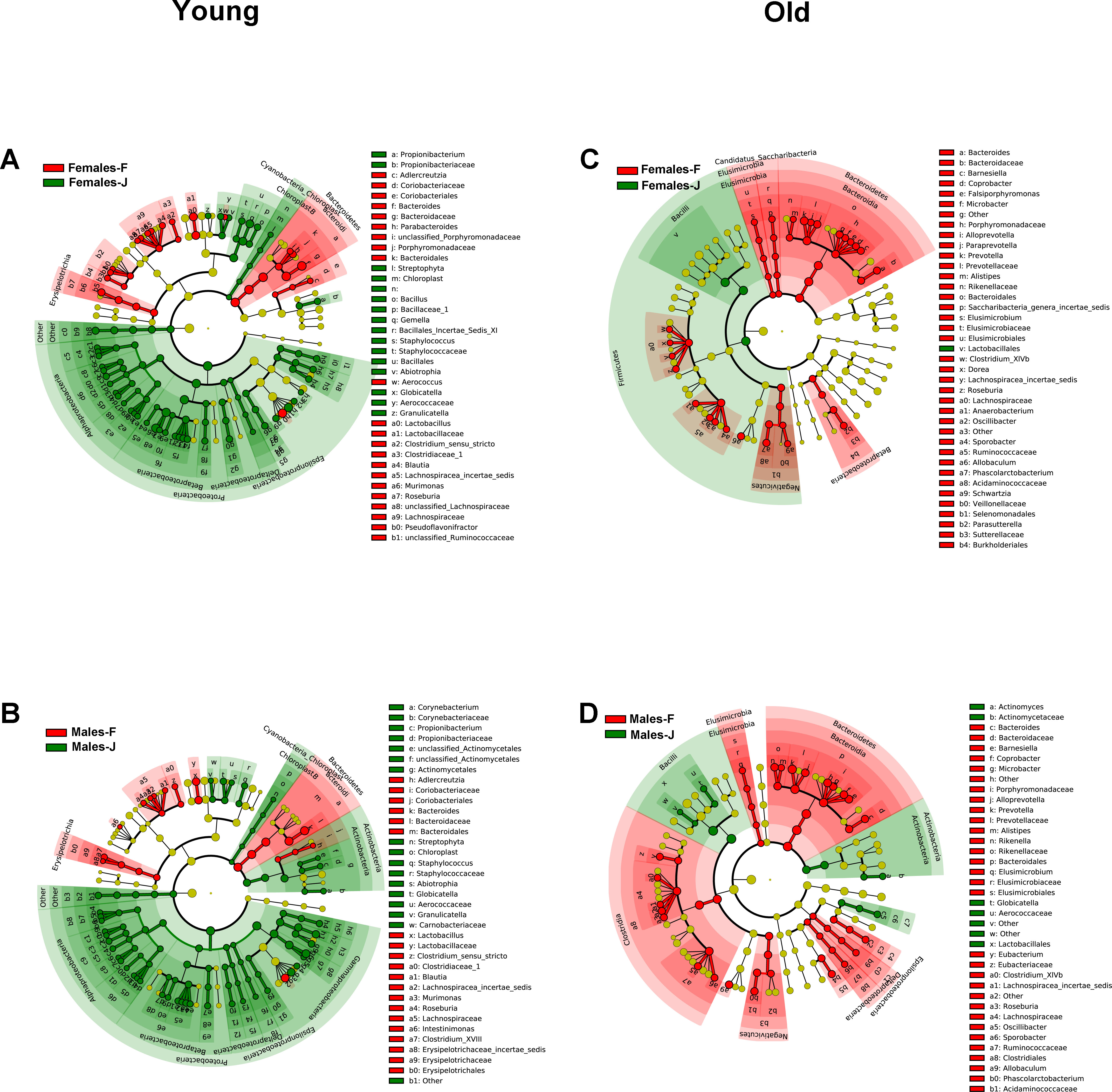


**Figure S3. Microbial taxa that are Markedly different between fecal and jejunal samples regardless of age and sex**. (A-B) Microbial taxa of females and males in young animals, respectively. (C-D) Microbial taxa of females and males in old animals, respectively. In this experiment, Mice purchased Hunan, n = 9–10 per group, F stands for fecal samples; J stands for jejunal samples. Each cladogram represents all taxa detected at the phylum to genus level. The size of the circle corresponds to the population of each taxon.

**Table S6. ANOSIM of gut microbiota composition of fecal samples from middle-aged rats, where individuals were grouped by F-Sham, OVX, OVX+E2, M-Sham, CAS and CAS+T.**

| Samples | Groups | R | *P*.value | Dissimilarity | Permutation |
| --- | --- | --- | --- | --- | --- |
| Feces | F-Sham VS OVX | 0.38 | 0.01 | Bray-curtis | 999 |
|  | OVX VS OVX+E2 | 0.74 | 6.00x10^-3^ | Bray-curtis | 999 |
|  | F-Sham VS OVX+E2 | 0.94 | 9.00x10^-3^ | Bray-curtis | 999 |
|  | M-Sham VS CAS | 0.90 | 6.00x10^-3^ | Bray-curtis | 999 |
|  | CAS VS CAS+T | 0.03 | 0.36 | Bray-curtis | 999 |
|  | M-Sham VS CAS+T | 0.82 | 2.00x10^-3^ | Bray-curtis | 999 |
|  | F-Sham VS M-Sham | 0.44 | 0.01 | Bray-curtis | 999 |
| jejunum | F-Sham VS OVX | 0.31 | 0.04 | Bray-curtis | 999 |
|  | OVX VS OVX+E2 | 0.06 | 0.33 | Bray-curtis | 999 |
|  | F-Sham VS OVX+E2 | 0.55 | 0.02 | Bray-curtis | 999 |
|  | M-Sham VS CAS | 0.37 | 0.04 | Bray-curtis | 999 |
|  | CAS VS CAS+T | 0.47 | 0.02 | Bray-curtis | 999 |
|  | M-Sham VS CAS+T | 0.2 | 0.09 | Bray-curtis | 999 |
|  | F-Sham VS M-Sham | -0.05 | 0.61 | Bray-curtis | 999 |

n = 5–6 per group, F stands for fecal samples; J stands for jejunal samples.

**Table S7. PERMANOVA of gut microbiota composition of fecal samples from middle-aged rats, where individuals were grouped by F-Sham, OVX, OVX+E2, M-Sham, CAS and CAS+T.**

| Samples | Groups | Df | SumsOfSqs | F.Model | R2 | *P*.value |
| --- | --- | --- | --- | --- | --- | --- |
| Feces | F-Sham VS OVX | 1 | 0.39 | 2.02 | 0.20 | 9.00x10^-3^ |
|  | OVX VS OVX+E2 | 1 | 0.54 | 3.33 | 0.29 | | 8.00x10^-3^ | | --- | |
|  | F-Sham VS OVX+E2 | 1 | 0.93 | 5.66 | 0.41 | 6.00x10^-3^ |
|  | M-Sham VS CAS | 1 | 0.72 | 4.90 | 0.38 | 0.01 |
|  | CAS VS CAS+T | 1 | 0.15 | 1.33 | 0.11 | 0.31 |
|  | M-Sham VS CAS+T | 1 | 0.70 | 4.52 | 0.33 | 0.04 |
|  | F-Sham VS M-Sham | 1 | 0.42 | 2.34 | 0.23 | 8.00x10^-3^ |
| jejunum | F-Sham VS OVX | 1 | 0.44 | 2.46 | 0.23 | 0.04 |
|  | OVX VS OVX+E2 | 1 | 0.22 | 1.33 | 0.14 | 0.27 |
|  | F-Sham VS OVX+E2 | 1 | 0.72 | 3.87 | 0.33 | 0.02 |
|  | M-Sham VS CAS | 1 | 0.33 | 3.21 | 0.29 | 0.04 |
|  | CAS VS CAS+T | 1 | 0.81 | 4.63 | 0.34 | 0.02 |
|  | M-Sham VS CAS+T | 1 | 0.42 | 2.25 | 0.20 | 0.06 |
|  | F-Sham VS M-Sham | 1 | 0.09 | 0.60 | 0.07 | 0.82 |

n = 5–6 per group, F stands for fecal samples; J stands for jejunal samples.

**
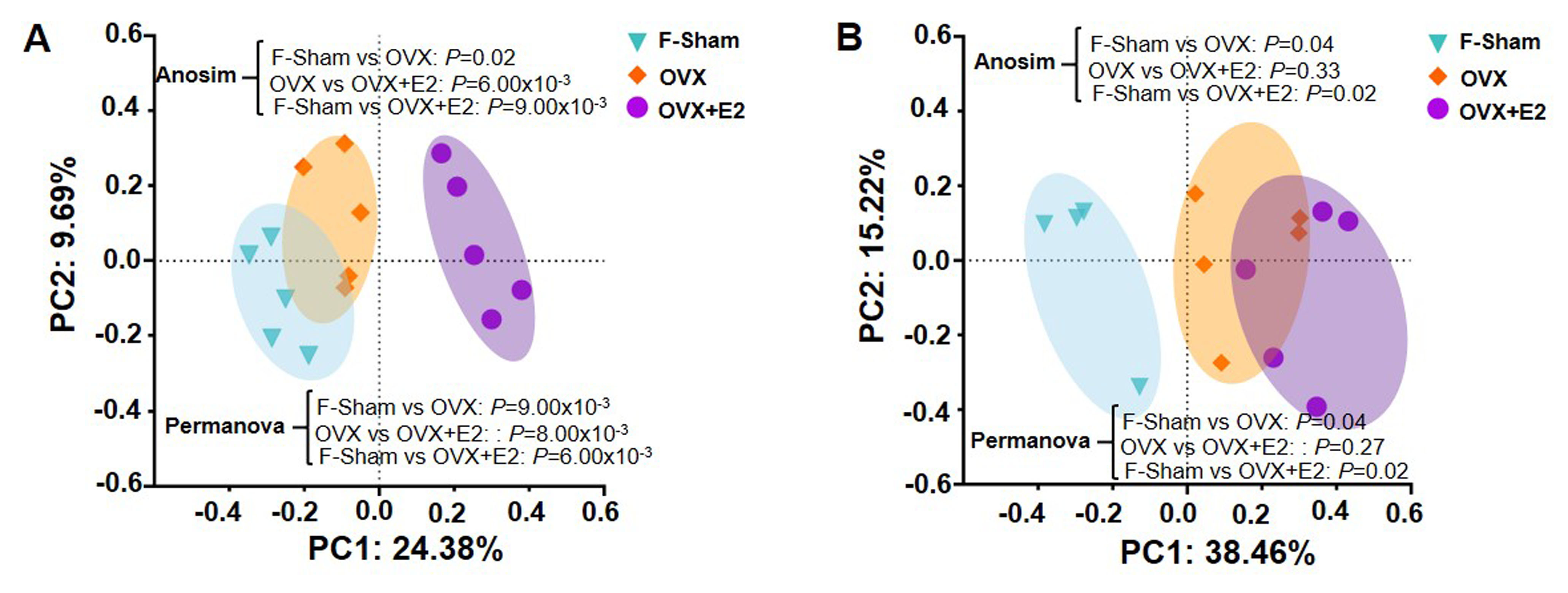
**

**Figure S4. The effect of gonadectomy on gut microbiota in middle-aged female rats.** (A) in fecal samples, principal coordinate analysis (PCoA) plot of Bray-Curtis distances of the microbial communities (B) In jejunum samples, the PCoA analysis of Bray-Curtis distances of the microbial communities. *P* values from ANOSIM and PERMANOVA are shown. In this experiment, individuals were grouped by F-Sham, OVX, OVX+E2, . n = 5–6 per group.

**
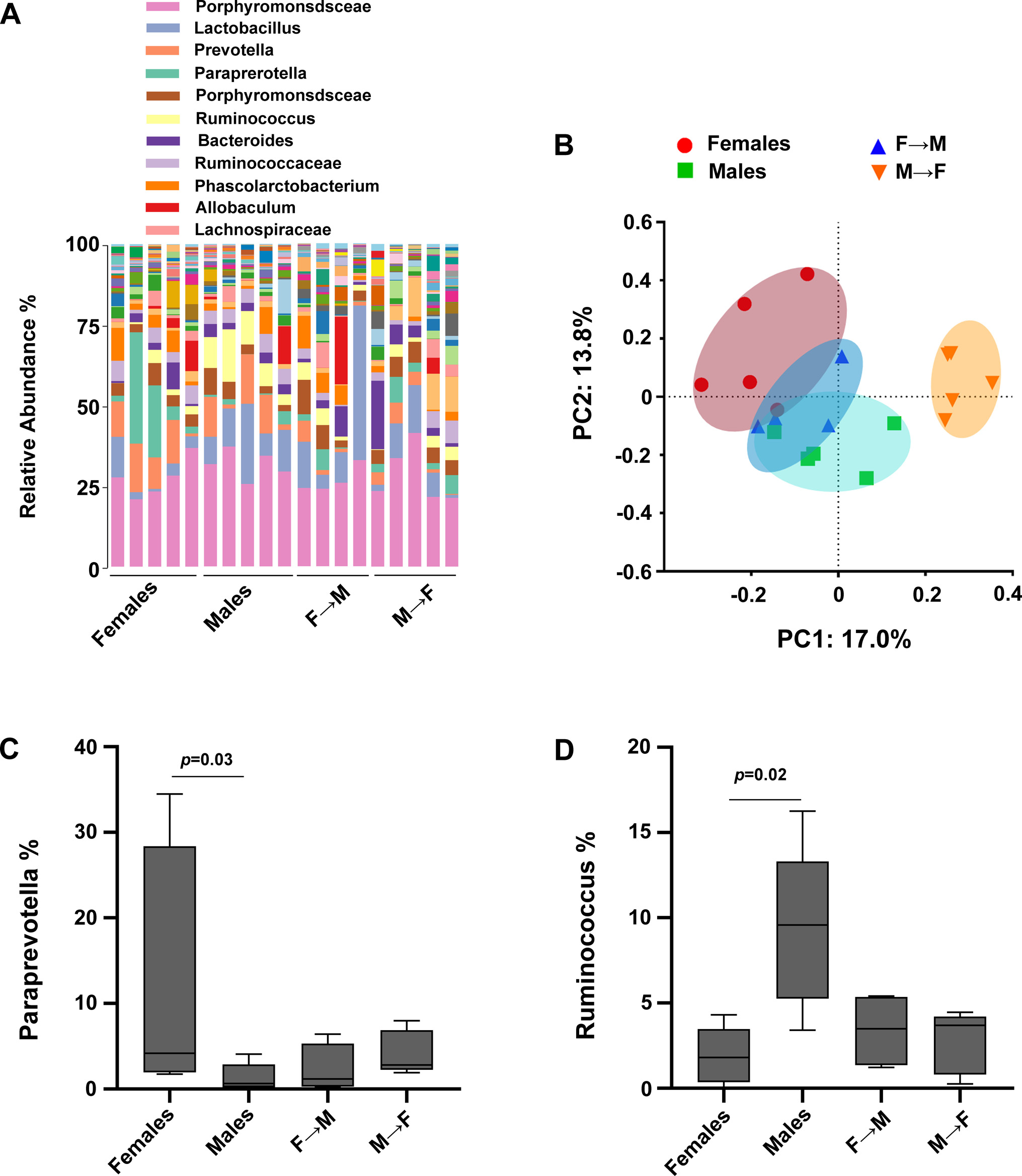
**

**Figure S5. The gut microbiota of receptors was intervented by donors in fecal microbiota transplantation (FMT)** **treatment.** (A) The relative abundance per individual for fecal samples from middle-aged rats. (B) PCoA plot. (C-D) Relative abundances distributions per group for the taxa (at genus level). In the experiment, individuals are grouped by Females, Males, F→M (Fecal microbiota from female rats was transplanted into male rats) and M→F (Fecal microbiota from male rats was transplanted into female rats). n = 4-5 per group, *P* values from Mann–Whitney U test. shown for the bacterial taxonomic rank of genus.

**Table S8. ANOSIM of gut microbiota composition of fecal samples from middle-aged rats, where individuals are grouped by Females, Males, F→M and M→F.**

| Groups | R | *P*.value | Dissimilarity | Permutation |
| --- | --- | --- | --- | --- |
| Females VS Males | 0.53 | 8.00x10^-3^ | Bray-curtis | 999 |
| Females VS F→M | 0.29 | 0.07 | Bray-curtis | 999 |
| Females VS M→F | 0.80 | 0.02 | Bray-curtis | 999 |
| Males VS F→M | 0.61 | 5.00x10^-3^ | Bray-curtis | 999 |
| Males VS M→F | 0.64 | 6.00x10^-3^ | Bray-curtis | 999 |
| F→M VS M→F | 0.59 | 0.01 | Bray-curtis | 999 |

n = 4–5 per group

**Table S9. PERMANOVA of gut microbiota composition of fecal samples from middle-aged rats, where individuals are grouped by Females, Males, F→M and M→F.**

| Groups | Df | SumsOfSqs | F.Model | R2 | *P*.value |
| --- | --- | --- | --- | --- | --- |
| Females VS Males | 1 | 0.45 | 2.62 | 0.25 | 8.00x10^-3^ |
| Females VS F→M | 1 | 0.34 | 1.66 | 1.19 | 0.03 |
| Females VS M→F | 1 | 0.60 | 3.20 | 0.29 | 9.00x10^-3^ |
| Males VS F→M | 1 | 0.42 | 2.39 | 0.25 | 7.00x10^-3^ |
| Males VS M→F | 1 | 0.47 | 2.94 | 0.27 | 0.01 |
| F→M VS M→F | 1 | 0.45 | 2.38 | 0.25 | 0.01 |

n = 4–5 per group


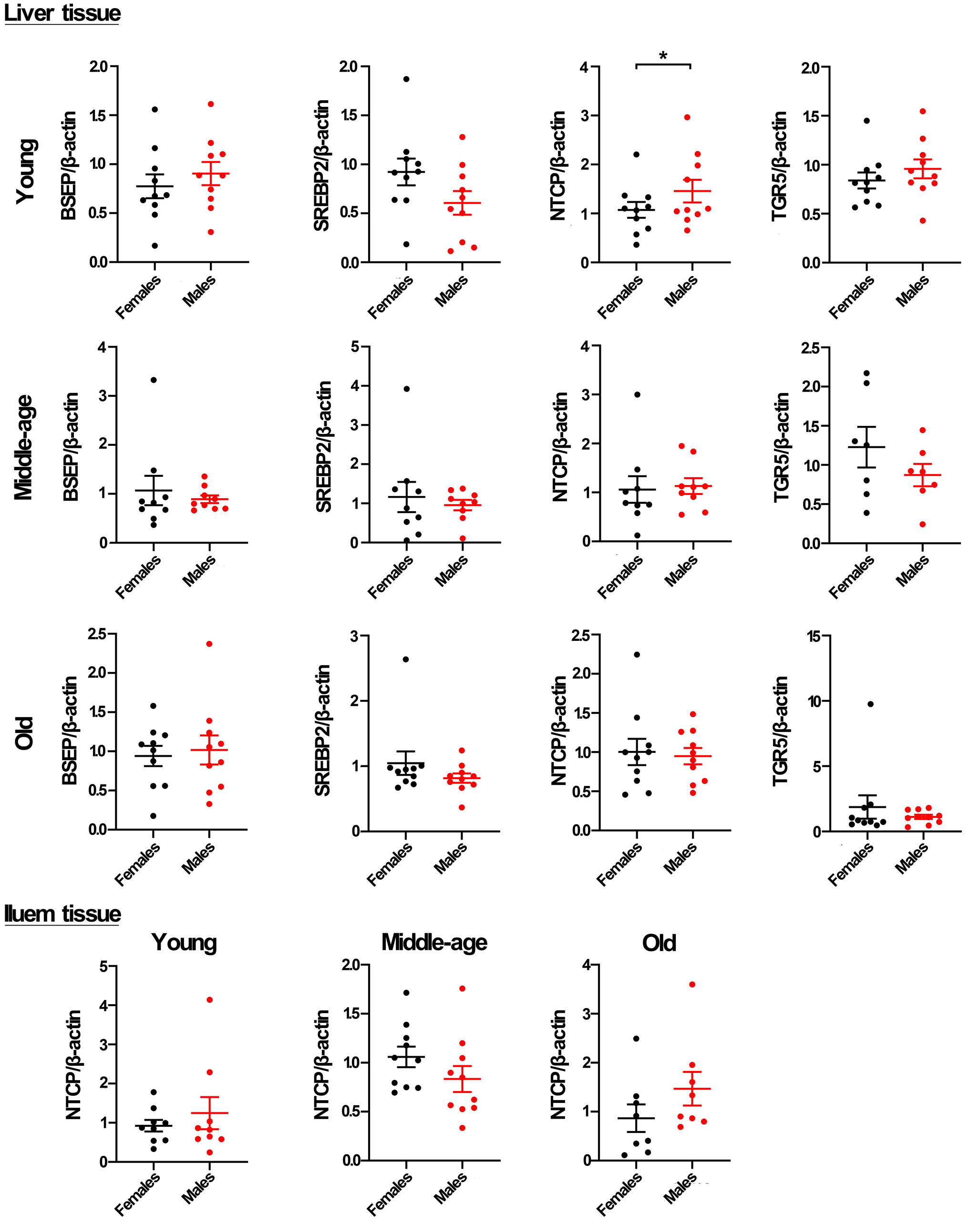


**Figure S6. Gender-specific expression of proteins related to bile acid signaling pathway in liver and ileum tissue from young, middle-aged, and old rats (females and males).** (A) BSEP, SREBP2, NTCP, and TGR5 protein abundances were detected by western blot. (B) Quantification of BSEP, SREBP2, NTCP, and TGR5 and these proteins expressions were normalized with β-actin (n = 9–10). Values are presented as mean ± SEM of three technical repetitions. Differences were assessed by student’s *t* test and denoted as follows: ***** *P* < 0.05; ****** *P* < 0.01; ******* *P* < 0.001; ns *P* > 0.05. Three biological replications were performed and all results were similar.


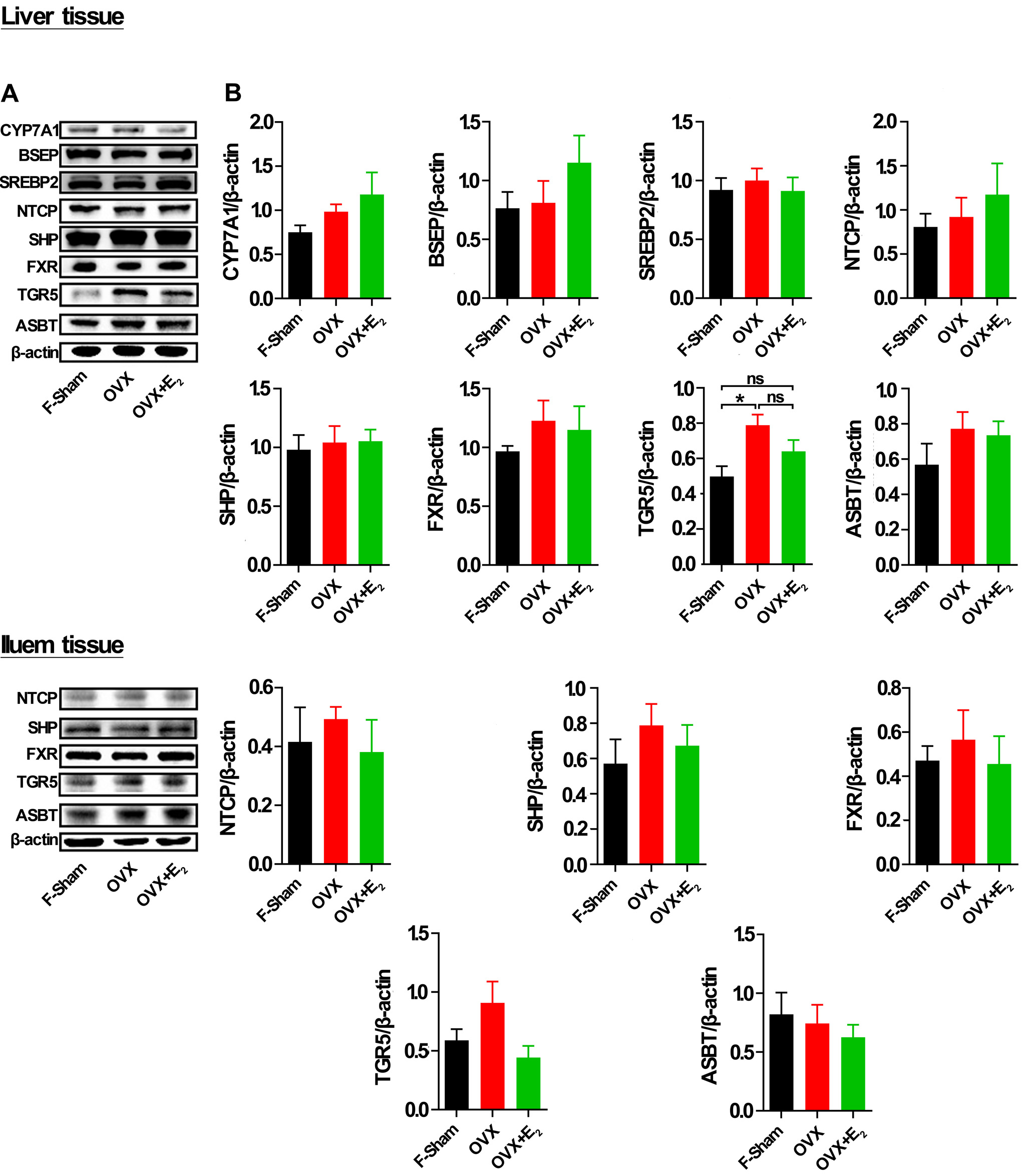


**Figure S7. The expression of proteins in the bile acid signaling pathway in liver and ileum tissue from middle-aged female rats.** Individuals were grouped by by Sham , OVX and OVX+E2. (A) CYP7A1, BSEP, SREBP2, NTCP, SHP, FXR, TGR5 and ASBT protein abundances were detected by Western blot. (B) Quantification of CYP7A1, BSEP, SREBP2, NTCP, SHP, FXR, TGR5 and ASBT and these proteins expressions were normalized with β-actin (n = 5–6). Values are presented as mean ± SEM of three technical repetitions. Differences were assessed by student’s *t* test and denoted as follows: ***** *P* < 0.05; ****** *P* < 0.01; ******* *P* < 0.001; ns *P* > 0.05. Three biological replications were performed and all results were similar.


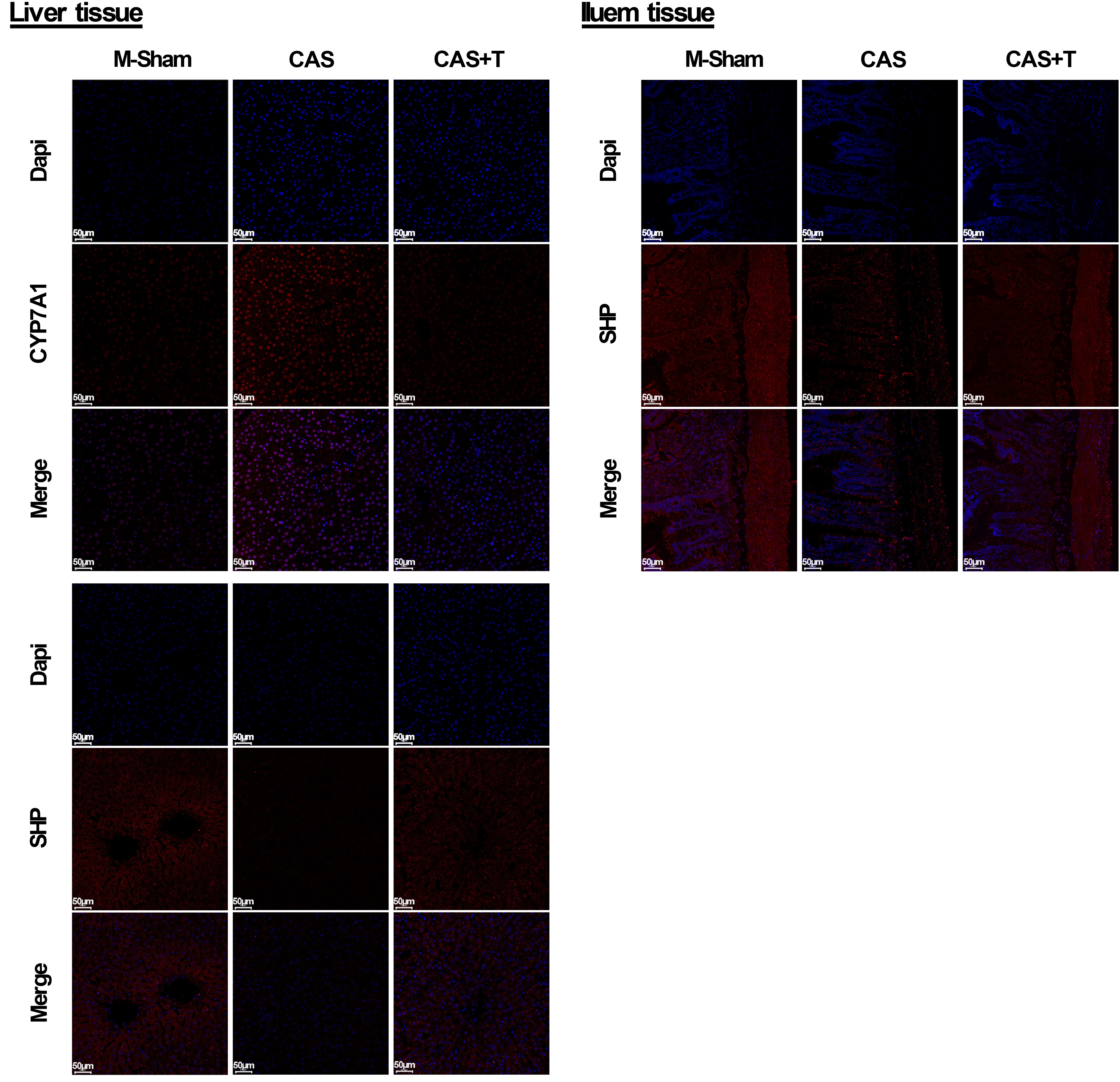


**Figure S8 Immunofluorescent analysis of CYP7A1 and SHP (red) were performed in liver and ileum tissue from middle-aged male rats. Individuals were grouped by Sham, CAS, CAS+T**. Nuclei were stained with DAPI (blue). Scale bar = 50 µm. three technical repetitions and three biological replications were performed and all results were similar.

**Table S10 Primers for quantitative real-time PCR (qRT-PCR)**

| **mRNA** | **Primer** | **Sequence** | **Accession Number** |
| --- | --- | --- | --- |
| *β-ACTIN*  *GAPDH*  *TGR5*  *CYP7A1*  *FXR*  *BSEP*  *NTCP*  *ASBT*  *SHP*  *SREBP2* | Forward  Reverse  Forward  Reverse  Forward  Reverse  Forward  Reverse  Forward  Reverse  Forward  Reverse  Forward  Reverse  Forward  Reverse  Forward  Reverse  Forward  Reverse | agaagagctatgagctgcctgacg  tggatgccacaggattccataccc  AGAGACAGCCGCATCTTCTTGTG  GATACGGCCAAATCCGTTCACACC  tgcttcgaggaagacccaagag  tcagtgctgcattggctactgg  TCATTACACTACTTCTGCGAAGGC  AGAAAGTGAACACAGAGCATCTCC  aggccatgttccttcgttcagc  tcatcggagatgccgctctttc  tctgacacatgaccctccactg  acaagcacgtcattgtccttgc  TCTCTTCCAACTCAATCCAAGCTG  AAAGTGGCCCAATGACTTCAGG  agatgtgggttgactcaggaacg  cgcaaccagagaaatgccaatgc  gcactatcctcttcaacccagatg  gctccaggacttcacacaatgc  tgtactgtcactggagtcaggttc  agcagtagagtcggcatcatcc | [NM_031144.2](about:blank)  NM_017008.3  [NM_177936.1](about:blank)  NM_012942.1  NM_021745.1  NM_031760.1  [NM_017047.1](https://quantprime.mpimp-golm.mpg.de/minimal.php?page=transcripts&subaction=geneinfo&identifier=NM_017047.1&organismid=1067)  NM_017222.2  [NM_057133.1](about:blank)  NM_001033694.1 |

**Table S11 Antibodies used in Western Blot**

| **Name** | **source** | **Working Dilution** |
| --- | --- | --- |
| Rabbit Anti-TGR5/GPBAR1 Antibody  Rabbit Anti-CYP7A1 Antibody  SHP2 Rabbit Polyclonal Antibody  Anti-ASBT/SLC10A2 Antibody  SLC10A7 Antibody (NTPC)  ABCB11 Antibody (BSEP)  SREBP2 Antibody  NR1H4 Antibody (FXR)  ACTB Rabbit mAb  Goat anti-Rabbit IgG | Novus cat.# NBP2-23669SS  Bioss cat.# bs-21430R  Proteintech cat.# 20145-1-AP  HUABIO cat. #ER1903-97  Affinity cat.# DF4515  Affinity cat.# DF9278  Affinity cat.# DF7601  Affinity cat.# DF12511  ABclonal cat.# AC026  HUABIO cat.# HA1001 | 1:500  1:500  1:500  1:500  1:500  1:1000  1:1000  1:500  1:50000  1:5000 |
